# DAB2IP loss in luminal a breast cancer leads to NF-**κ**B–associated aggressive oncogenic phenotypes

**DOI:** 10.1172/jci.insight.171705

**Published:** 2024-12-06

**Authors:** Angana Mukherjee, Rasha T. Kakati, Sarah Van Alsten, Tyler Laws, Aaron L. Ebbs, Daniel P. Hollern, Philip M. Spanheimer, Katherine A. Hoadley, Melissa A. Troester, Jeremy M. Simon, Albert S. Baldwin

**Affiliations:** 1 UNC Lineberger Comprehensive Cancer, University of North Carolina at Chapel Hill, Chapel Hill, North Carolina, USA.; 2 Department of Pathology and Laboratory Medicine and; 3 Division of Surgical Oncology, University of North Carolina School of Medicine, Chapel Hill, North Carolina, USA.; 4 UNC Gillings School of Global Public Health, University of North Carolina at Chapel Hill, Chapel Hill, North Carolina, USA.; 5 The Salk Institute, La Jolla, California, USA.; 6 Department of Genetics and; 7 UNC Neuroscience Center, University of North Carolina School of Medicine, Chapel Hill, North Carolina, USA.; 8 Department of Data Science, Dana-Farber Cancer Institute and Department of Biostatistics, Harvard T.H. Chan School of Public Health, Boston, Massachusetts, USA.; 9 Department of Biology, University of North Carolina at Chapel Hill, North Carolina, USA.

**Keywords:** Oncology, Breast cancer, NF-kappaB, Tumor suppressors

## Abstract

Despite proven therapy options for estrogen receptor–positive (ER^+^) breast tumors, a substantial number of patients with ER^+^ breast cancer exhibit relapse with associated metastasis. Loss of expression of RasGAPs leads to poor outcomes in several cancers, including breast cancer. Mining the The Cancer Genome Atlas (TCGA) breast cancer RNA-Seq dataset revealed that low expression of the RasGAP *DAB2IP* was associated with a significant decrease in relapse-free survival in patients with Luminal A breast cancer. Immunostaining demonstrated that DAB2IP loss occurred in grade 2 tumors and higher. Consistent with this, genes upregulated in *DAB2IP*-low Luminal A tumors were shared with more aggressive tumor subtypes and were associated with proliferation, metastasis, and altered ER signaling. Low DAB2IP expression in ER^+^ breast cancer cells was associated with increased proliferation, enhanced stemness phenotypes, and activation of IKK, the upstream regulator of the transcription factor NF-κB. Integrating cell-based ChIP-Seq with motif analysis and TCGA RNA-Seq data, we identified a set of candidate NF-κB target genes upregulated with loss of DAB2IP linked with several oncogenic phenotypes, including altered RNA processing. This study provides insight into mechanisms associated with aggressiveness and recurrence within a subset of the typically less aggressive Luminal A breast cancer intrinsic subtype.

## Abstract 2

This study offers new insight into a subset of Luminal A breast cancer shown to be at higher risk of recurrence with loss of DAB2IP.

## Introduction

Since the 2000s, the incidence of female breast cancer has increased by approximately 0.5% per year (1). In 2021, breast cancer accounted for 12% of all new yearly cancer cases, thereby becoming the most commonly diagnosed cancer among women worldwide (2). It is estimated that approximately 43,000 breast cancer deaths will occur in the United States in 2024 (3). Breast cancer is a collection of heterogeneous diseases, exhibiting varied responses to different clinical treatment approaches (1). Although early diagnosis often results in a favorable outcome, clinical and molecular features vary across the intrinsic breast tumor subtypes (4).

Global gene expression studies have established 5 breast cancer subtypes, namely Luminal A, Luminal B, Basal-like, HER2-enriched, and Claudin-low (5). Whereas Basal-like and HER2-enriched cancers exhibit little to no expression of estrogen receptor (ER) and ER-related genes, both Luminal A and Luminal B subtypes are characterized by expression and activity of ER (5). Furthermore, Basal-like and HER2-enriched cancers have generally more aggressive phenotypes than luminal subtypes (5). The Luminal A subtype, which constitutes about 50%–60% of all invasive breast cancers, is characterized by low nuclear pleomorphism, low mitotic activity, low histological grade, and therefore a generally good prognosis (4–6). The Luminal B subtype constitutes about 20% of all invasive breast cancers and has a higher proliferation index, more aggressive phenotypes, higher histologic grade, worse prognosis, and increased relapse rates when compared with Luminal A (4, 5). Patients with Luminal B breast cancers have rates of overall survival that are similar to the high-risk Basal-like and HER2-enriched subtypes (4, 5). Unlike Luminal A tumors, which generally respond well to endocrine therapies, Luminal B tumors exhibit a better response to neoadjuvant chemotherapy, although patients with these tumors have higher recurrence rates compared with patients with Luminal A tumors (4, 5). Nevertheless, even though Luminal A breast cancer has been shown to be of low risk overall, 10%–13% of these patients exhibit metastatic relapse (6). Hence, there is a major need for a better understanding of the molecular basis driving therapeutic resistance and recurrence in ER^+^ subtypes, particularly for the Luminal A subtype.

Although molecular subtypes are robust predictors of outcomes, patient-to-patient variability in response to therapy and outcomes exists even within molecular subtypes. It is therefore essential to understand relevant biological differences within subtypes, which may further divide into subgroups, to identify patients at risk for reduced response to therapy and/or worse outcomes. For instance, Olsen et al. showed that loss of expression of the RasGAP tumor suppressor *DAB2IP* — often along with loss of another RasGAP, *RASAL2* — promotes poorer outcome in approximately 50% of Luminal B breast cancer, which is associated with Ras signaling pathways and NF-κB transcription factor activity (7). Here, we explored Luminal A breast cancer and showed that low *DAB2IP* expression leads to poorer outcomes, similar to what is described for patients with Luminal B breast cancer. Interestingly, loss of DAB2IP expression in ER^+^ tumors, including Luminal A tumors, occurs predominantly in grade 2 or higher tumors. We investigated the effect of low *DAB2IP* on the gene expression profile of The Cancer Genome Atlas (TCGA) patients with breast cancer, identifying genes upregulated in Luminal A tumors with low *DAB2IP* expression, which include genes associated with proliferation, epithelial-mesenchymal transition (EMT), and metastasis that are also enriched in Luminal B and HER2-enriched cancers, irrespective of their *DAB2IP* status. Additionally, analysis of the TCGA-derived data suggests that loss of *DAB2IP* expression leads to an altered ER transcriptional response. Furthermore, *DAB2IP* knockdown promoted proliferation in Luminal A breast cancer cells and promoted stemness in both Luminal A and Luminal B cells. Furthermore, we showed that loss of *DAB2IP* expression led to activation of IKK, the upstream kinase in the NF-κB pathway. In this regard, we identified a candidate set of NF-κB target genes differentially expressed between *DAB2IP-*low and -high Luminal A tumors that are associated with aggressive cancers, including some involved in RNA splicing. In addition, integrating ChIP-Seq performed in a Luminal A cell line with DAB2IP knockdown and TCGA RNA-Seq data, we propose a set of RELA-, RELB-, and NFKB2-regulated genes that are associated with low *DAB2IP* and aggressive oncogenic phenotypes. Our results demonstrate the consequence of loss of *DAB2IP* in ER^+^ breast cancer, specifically focused on Luminal A breast cancer, and provide insight into underlying mechanisms that lead to the aggressiveness of this subset of tumors.

## Results

### Luminal A breast tumors with low DAB2IP expression are associated with poor survival and an increased risk of recurrence.

**Figure 1:**
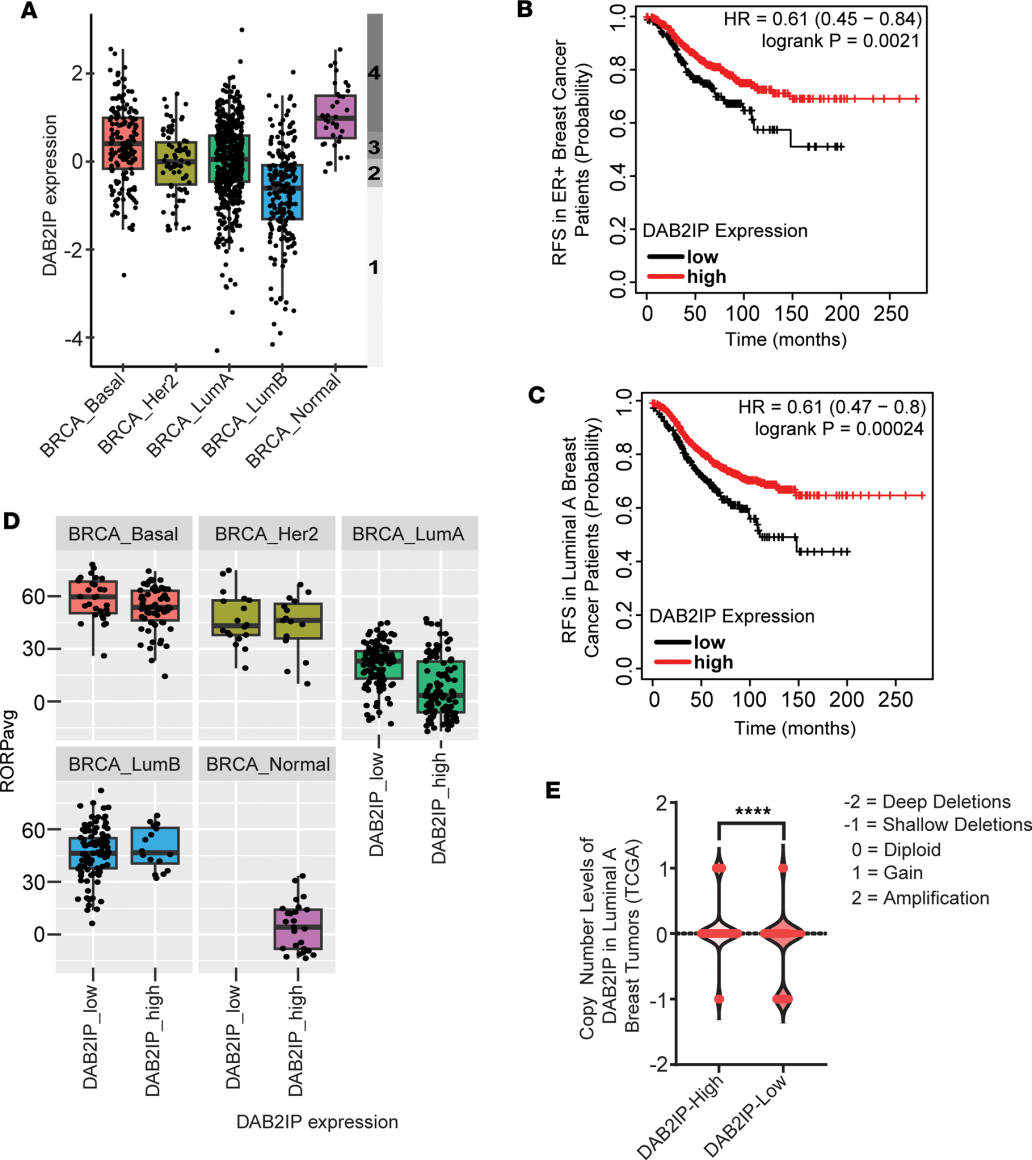
Low *DAB2IP* expression in Luminal A breast cancer subtype is associated with poorer survival. (**A**) TCGA-breast cancer RNA-Seq data (*n* = 1,082) was retrieved from cBioPortal, and tumors were divided into quartiles based on *DAB2IP* expression. Shaded numbers (nos. 1–4) indicate the quartiles, 1 being the lowest 25% (*DAB2IP*-low) and 4 the highest 25% (*DAB2IP*-high). (**B** and **C**) Relapse-free survival curves for patients with ER^+^ breast cancer and patients with Luminal A breast cancer based on *DAB2IP* expression were plotted using the Kaplan-Meier Plotter website. DAB2IP-225020_at probe was used to generate the curves (ER^+^: *n* = 877; Luminal A: *n* = 952). (**D**) The PAM50 based ROR-P score between high and low *DAB2IP* was determined for each breast cancer subtype (Luminal A: *P* = 3.064 × 10^–10^). (**E**) Copy number alterations ranging from –2 to 2 were plotted based on *DAB2IP*-high/low status in Luminal A TCGA breast cancer cohort (****P < 0.0001). Data were analyzed using unpaired Student’s *t* test.

To analyze *DAB2IP* expression across breast cancer subtypes, we utilized TCGA breast cancer RNA-Seq data from 1,082 breast tumors (8, 9). We then stratified all tumors into quartiles based on *DAB2IP* expression and defined the first quartile (lowest 25%) as “*DAB2IP*-low” and the fourth quartile (highest 25%) as “*DAB2IP*-high”. Our analysis revealed that approximately 50% of Luminal B tumors exhibit low *DAB2IP* expression (Figure 1A), consistent with the previous report of Olsen et al. (7). Interestingly, we find that *DAB2IP* expression is also reduced in approximately 25%–30% of patients with Luminal A tumors (Figure 1A). Analysis of the METABRIC cohort produced similar observations in both luminal subtypes (Supplemental Figure 1A; supplemental material available online with this article; https://doi.org/10.1172/jci.insight.171705DS1).

We first studied the association between survival rate and low *DAB2IP* expression in patients with ER^+^ cancer broadly and then specifically in patients with Luminal A breast cancer. Extending beyond the intrinsic subtypes, we found that patients with ER^+^ tumors that have low *DAB2IP* expression exhibit significantly poorer survival (Figure 1B; hazard ratio [HR], 0.61; *P* = 0.0021). Furthermore, Kaplan-Meier analysis showed that, for patients with Luminal A tumors, low *DAB2IP* expression was associated with a significant decrease in relapse-free survival time compared with those with high *DAB2IP* (Figure 1C; HR, 0.61; *P* = 0.00024).

The Prediction Analysis of Microarray 50–based (PAM50-based) risk of recurrence (ROR) score (Supplemental Table 1) is an established predictor of 10-year distant recurrence in patients with breast cancer (10, 11); therefore, we analyzed the ROR-Proliferation score (ROR-P) between *DAB2IP*-high and *DAB2IP*-low expression groups for each breast tumor subtype. While we did not find any difference in other subtypes relative to high and low DAB2IP expression, for Luminal A tumors, we found that the risk of distant recurrence score was significantly higher in *DAB2IP*-low tumors (Figure 1D) as compared with *DAB2IP*-high tumors. Since copy number alterations (CNAs) are generally associated with cancer progression (12), we then examined the number of CNAs based on *DAB2IP* status in the Luminal A breast cancer TCGA cohort. Interestingly, *DAB2IP*-low Luminal A tumors exhibited more CNAs, in particular shallow deletions, which are heterozygous deletions, when compared with the *DAB2IP*-high group (Figure 1E).

Taken together, these results suggest that, alongside Luminal B, a subset of Luminal A tumors also exhibits loss of *DAB2IP*, which in turn is associated with poor survival and a significantly higher recurrence score in these tumors. Furthermore, low *DAB2IP* expression is associated with advanced, more aggressive Luminal A breast cancers, as indicated by more CNAs.

### Stratification of tumor subtypes based on DAB2IP expression.

**Figure 2:**
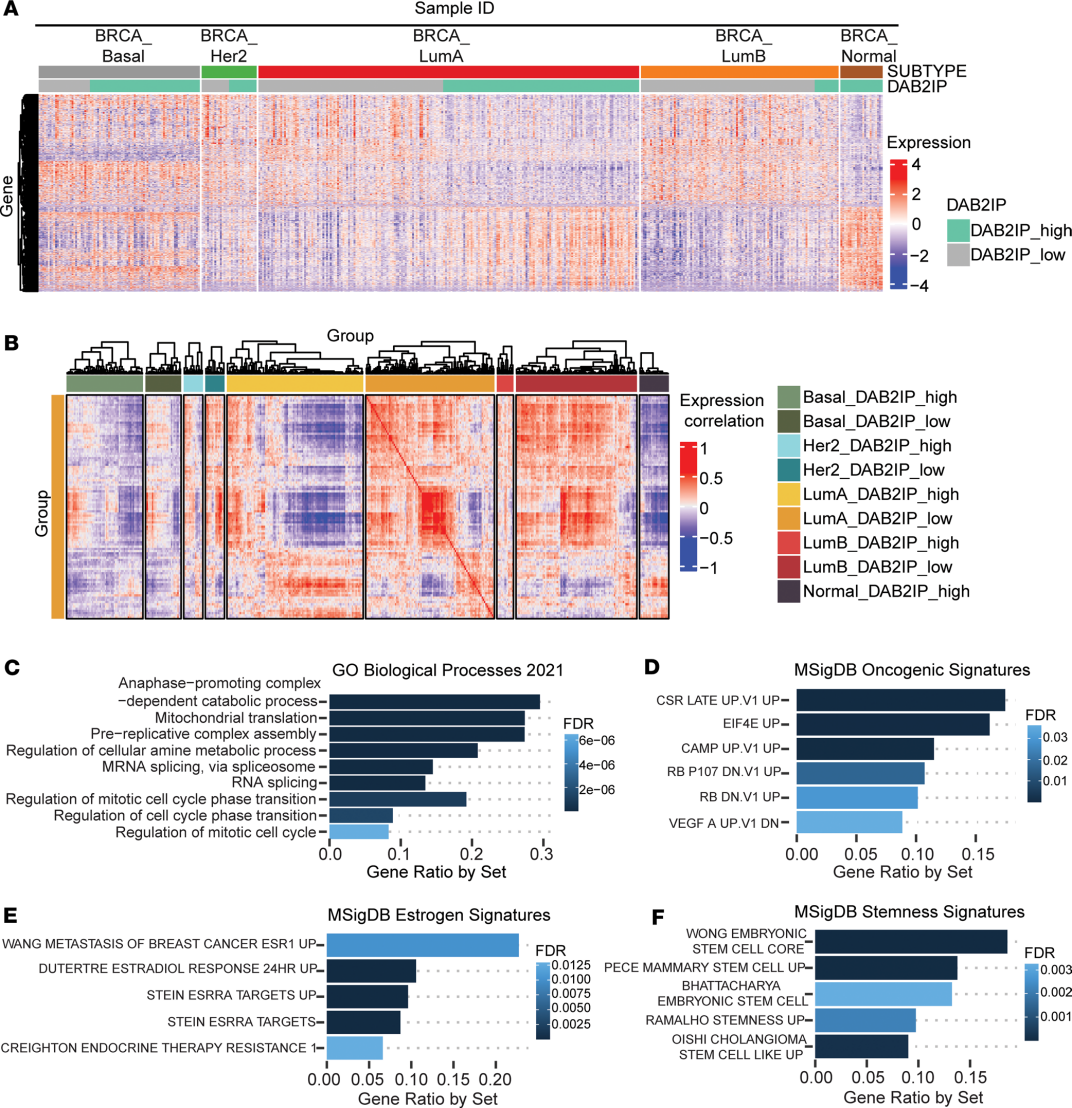
Gene expression profiling based on *DAB2IP* status revealed distinct clusters of differentially expressed genes in Luminal A subtype. (**A**) DESeq analysis of TCGA Luminal A breast cancer RNA-Seq data was performed using quartile-based cutoffs to divide patients into high/low *DAB2IP* groups. The expression map shows differentially expressed genes (DEGs) between *DAB2IP* high/low Luminal A subtype, clustered across all breast cancer subtypes (*P*_adj_ < 1 × 10^–5^). (**B**) Pearson correlation expression map showing positive correlation between Luminal A *DAB2IP*-low group, HER2 (high and low), and Luminal B (high and low) subtypes. (**C** and **D**) DEGs upregulated with low *DAB2IP* in Luminal A tumors were subjected to gene enrichment analysis using GO biological processes and oncogenic pathway activation gene sets (FDR > 0.05). (**E** and **F**) Upregulated DEGs in *DAB2IP*-low Luminal A tumors were subjected to enrichment analysis using MSigDB-curated gene sets to show the overlap with ESSRA targets, estradiol-associated gene sets, gene sets associated with endocrine therapy resistance, and stemness (FDR > 0.05). Gene expression associations with *DAB2IP* class were analyzed using unpaired Student’s *t* test.

To examine the effect of low *DAB2IP* expression on the global transcriptome of all ER^+^ breast tumors, we performed differential expression analysis, comparing the highest and lowest quartiles of *DAB2IP* expression among nonbasal ER^+^ tumors across all subtypes (*n* = 320). We observed 1,018 genes significantly differentially expressed by *DAB2IP* level (465 upregulated and 553 downregulated) that displayed distinctive expression patterns almost exclusively in Luminal A tumors (Supplemental Figure 1B). This observation led us to perform differential expression analysis, specifically comparing the highest and lowest quartiles of *DAB2IP* expression among Luminal A tumors and led us to clustering them across all subtypes (Figure 2A). We detected 1,120 genes significantly upregulated and 953 genes significantly downregulated according to *DAB2IP* level in Luminal A tumors (Figure 2A). We noticed that *DAB2IP*-low Luminal A tumors exhibited expression patterns of a subset of these differentially expressed genes (DEGs) that resembled those of Luminal B tumors; therefore, we quantified the similarity between Luminal A *DAB2IP*-low tumors with all other tumor subtypes using Pearson correlations of all DEGs. Interestingly, we found that Luminal A *DAB2IP*-low tumors correlated more closely with Luminal B and HER2 tumors, irrespective of their *DAB2IP* status (Figure 2B). These data indicate that Luminal A tumors with low *DAB2IP* expression exhibit differential gene expression patterns that more closely resemble Luminal B and HER-2–enriched phenotypes, which generally exhibit advanced stage and worse outcomes.

Next, we associated genes that were differentially expressed between *DAB2IP*-high and *DAB2IP*-low Luminal A tumors with biological and oncogenic functions by subjecting the upregulated and downregulated genes to functional Gene Set Enrichment Analysis (GSEA) enrichment analysis (13, 14). Cell cycle–related genes (Figure 2C and Supplemental Table 2) and other proliferative gene sets, such as those associated with loss of tumor suppressor Rb and upregulation of EIF4E (Figure 2D and Supplemental Table 2), were enriched among genes expressed more highly in *DAB2IP*-low Luminal A tumors. While we did not find upregulation of a classical ER-regulated gene signature in the *DAB2IP*-low Luminal A group, the enrichment analysis revealed enhanced ER-induced gene regulation associated with low-*DAB2IP* (Figure 2E and Supplemental Table 2). Estrogen regulated genes such as *GINS2*, *CDC6*, *CENPU*, and *BRIP1* (15, 16), along with others (Figure 2E and Supplemental Table 2), were highly expressed in the *DAB2IP*-low Luminal A tumors. Furthermore, genes associated with resistance to endocrine therapy, such as *SNRPE*, *CDKN3*, and *CCNB2* (17), were more highly expressed in the *DAB2IP*-low Luminal A tumors (Figure 2E and Supplemental Table 2). In addition, we also observed that genes contributing to cancer cell stemness, such as *CDKN3*, *BRIX1*, *TBCA*, *TMX2*, and others (18), were highly expressed in the Luminal A *DAB2IP*-low tumors compared with the *DAB2IP*-high Luminal A tumors (Figure 2F and Supplemental Table 2). In contrast, the genes that were downregulated in the *DAB2IP*-low Luminal A tumors were enriched for pathways associated with extracellular matrix organization and assembly, negative regulation of epithelial cell differentiation, and negative regulation of blood vessel morphogenesis (Supplemental Figure 1C and Supplemental Table 2).

We identified individual genes upregulated in *DAB2IP*-low Luminal A tumors that are known to play important roles in cancer progression and therapy resistance (Supplemental Table 3). For instance, *BIRC5* was upregulated in *DAB2IP*-low Luminal A tumors, and its expression has been proposed to drive the progression of breast and other cancers (19). We also identified *SRSF1*, an RNA-binding protein strongly linked with breast cancer progression and metastasis (20), to be upregulated in *DAB2IP*-low Luminal A tumors. Furthermore, cell cycle genes such as *CDK5*, *CCNA2*, *CCNB1*, and *CCNB2*, associated with tumor relapse and metastasis (21), are more highly expressed in *DAB2IP*-low Luminal A tumors. The epithelial cell adhesion molecule *EPCAM* — a transmembrane glycoprotein overexpressed particularly in tumor-initiating cells (TICs) in various cancers, including breast cancer — was also more highly expressed in *DAB2IP-*low Luminal A tumors (22).

Using the METABRIC dataset as an independent cohort, we identified DEGs between low and high *DAB2IP* expression quartiles in Luminal A and Luminal B subtypes (Supplemental Figure 1D). Similar to the TCGA findings described above, gene ontology (GO) analysis showed that the genes upregulated in the *DAB2IP*-low ER^+^ group are associated with cell proliferation (Supplemental Figure 1E and Supplemental Table 2). For example, pathway analysis reveals the association of highly expressed genes in *DAB2IP*-low ER^+^ tumors with STK33, a protumorigenic kinase that increases proliferation in breast cancer cells and has been associated with advanced colorectal and pancreatic malignancies (23). Interestingly, the genes more highly expressed in *DAB2IP-*low tumors also displayed enrichment of NF-κB signaling pathway genes, along with genes regulating a canonical hallmark of cancer progression, namely epithelial-to-mesenchymal transition (EMT) (Supplemental Figure 1F and Supplemental Table 2).

Taken together, Luminal A breast cancer with low *DAB2IP* expression is associated with increased cancer hallmark characteristics, including cell proliferation and metastasis, which may be due to increased NF-κB and enhanced ER signaling. Interestingly, analysis of METABRIC data revealed commonalities in the expression of genes that discriminated *DAB2IP-*high/low tumors that extended beyond the luminal subtypes (Supplemental Figure 1D), suggesting that the downstream effectors of low *DAB2IP* expression may not be restricted to ER^+^ tumors.

### DAB2IP loss is enriched in higher-grade and later-stage tumors.

**Figure 3:**
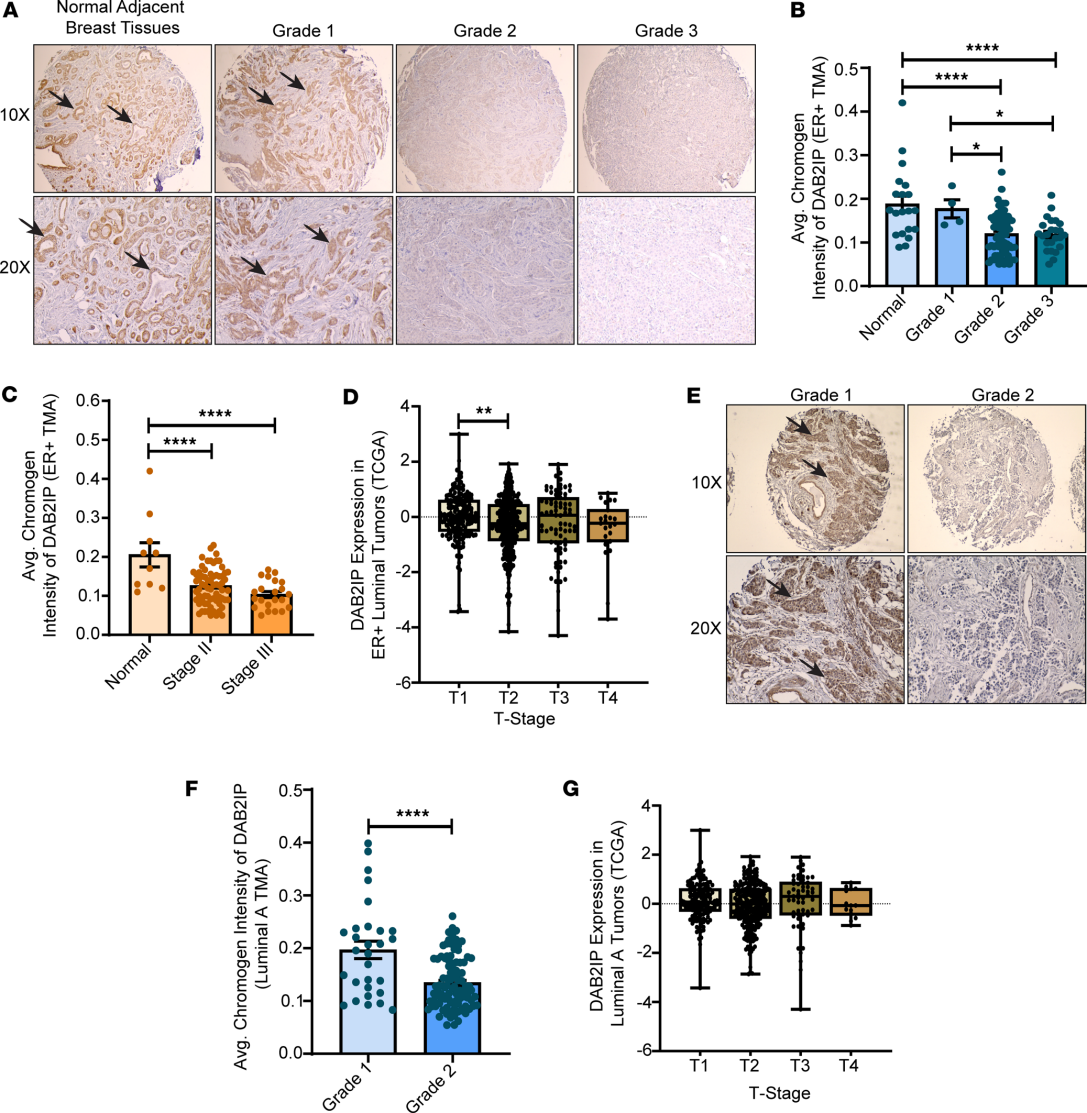
DAB2IP expression decreases with increasing tumor grade and stage in human ER^+^ and Luminal A–only breast cancer specimens. (**A**) Representative images show DAB2IP expression from IHC studies of 116 ER^+^ breast cancer specimens. Arrows indicate positive cells. Magnification, 10× (top), 20× (bottom). (**B** and **C**) Staining intensity of DAB2IP expression per specimen was quantified computationally and plotted by tumor grades and stage, respectively (grade 1 vs. grade 2, *P = 0.0222; grade 1 vs. grade 3, *P = 0.0259; normal vs. grade 2 and normal vs. grade 3, ****P < 0.0001; normal vs. stage II and normal vs. stage III, ****P < 0.0001). (**D**) *DAB2IP* expression in TCGA ER^+^ luminal patients was graphed according to the respective tumor stage (T1–T4) (**P = 0.0035). (**E**) Representative images show DAB2IP expression of 126 Luminal A breast cancer tissues with arrows indicating positively stained cells. Magnification, 10× (top), 20× (bottom). (**F**) DAB2IP expression intensity in Luminal A tumors quantified per specimen was graphed by tumor grades (****P < 0.0001). (**G**) *DAB2IP* expression in TCGA patients with Luminal A breast cancer was plotted according to the respective tumor stage. Data were analyzed using unpaired Student’s *t* test, and multiple comparisons were corrected with Dunnett’s test.

DAB2IP is inactivated in malignant lesions by multiple mechanisms, which include promoter hypermethylation by the EZH2-PRC2 complex; posttranscriptional silencing by microRNAs; degradation by SMURF1, SKP2, and FBW7; and phosphorylation by AKT1 (24). To extend and confirm the RNA studies, we tested DAB2IP protein expression and association with clinical grade by performing IHC staining on ER^+^ breast cancer tissue microarrays (TMAs). These TMAs consisted of 116 tumors that are broadly ER^+^ with different breast cancer pathologies and also included normal adjacent tissue (NAT) (Supplemental Table 4). Both NAT and grade 1 breast tumor tissues stained positively for DAB2IP (Figure 3, A and B). However, with progression to grades 2 and 3, we noted a significant decrease in DAB2IP expression in many of these tissues compared with normal and grade 1 (Figure 3, A and B, and Supplemental Figure 2A). We also stratified 112 of the above 116 tumors based on stage, the primary factor used clinically to assess the ROR, and stratified patients for treatment intensity. DAB2IP expression was significantly lower in overall stage II tumors (Figure 3C). Furthermore, broadly categorized stage III tumors had significantly lower DAB2IP expression compared with normal tumors (Figure 3C). Since low DAB2IP expression correlated with higher grades and stages, we next stratified 89 of the above 116 tumors based on their respective Ki67 index percentages, a proliferation marker for tumor cells. We found that, even though not significant, patients with higher grades displaying low DAB2IP had a higher Ki67 index percentage (Supplemental Figure 2B). TCGA breast cancer data also show a similar decrease in *DAB2IP* expression at the RNA level with increasing T-stage in patients with ER^+^ luminal breast cancer, particularly significant in T2 tumors (Figure 3D). In addition, we analyzed DAB2IP protein expression in 126 Luminal A–only tumors exhibiting different grades and stages (Supplemental Table 4). We found that, in comparison with grade 1 tumors, DAB2IP loss was significantly associated with grade 2 Luminal A tumors (Figure 3, E and F, and Supplemental Figure 2C). Furthermore, although not significant, TCGA Luminal A RNA-Seq data indicate lower *DAB2IP* expression in patients with T2 and T4 Luminal A breast cancer compared with normal tumors (Figure 3G).

Thus, high-grade (grades 2 and 3) and higher-stage (stages II and III) ER^+^ tumors, including high-grade Luminal A–only tumors (grade 2), are more likely to exhibit loss of DAB2IP expression as compared with NAT and low-grade and lower-stage tumors, supporting the TCGA RNA-Seq analysis on loss of expression of *DAB2IP* in ER^+^ tumors, specifically in Luminal A tumors.

### Loss of DAB2IP increases the proliferation and migration of Luminal A breast cancer cells.

**Figure 4:**
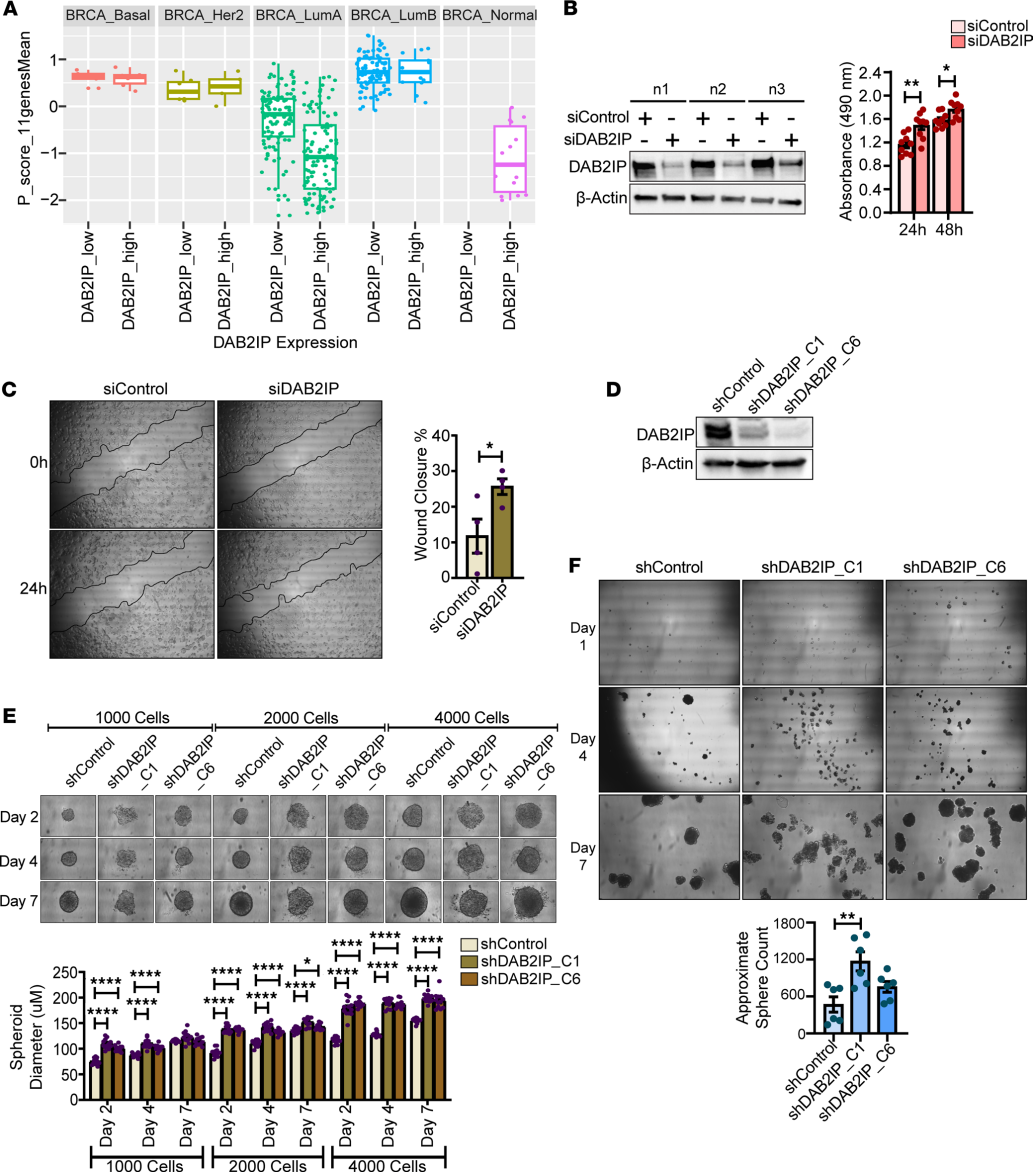
Loss of DAB2IP increases proliferation, migration, tumorspheroid, and tumorsphere formation in Luminal A cells. (**A**) Proliferation score distribution based on an 11-gene signature was analyzed across breast cancer subtypes by *DAB2IP* levels (Luminal A: *P* = 1.555 × 10^–11^). (**B**) Proliferation rate of T47D cells transfected with siRNA specific to DAB2IP or nontargeting control pool was examined using MTS assay (**P = 0.0025, *P = 0.0133) (*n* = 9). (**C**) After 24-hour transfection, a scratch-wound assay was performed on T47D cells (*P = 0.0391) (*n* = 4). (**D**) T47D cells were transduced with control shRNA or 2 different clones of DAB2IP-trageting shRNAs, and knockdown efficiency was determined by Western blot. (**E**) In total, 1,000; 2,000; and 4,000 shDAB2IP and shControl T47D cells were seeded for tumorspheroid assay, with images captured on days 2, 4, and 7 (*P = 0.041, ****P < 0.0001) (*n* = 3 and 4 measurements were taken per spheroid). (**F**) shDAB2IP and shControl T47D cells were plated for tumorsphere assays, with images taken on days 1, 4, and 7. Tumorsphere quantification was performed on day 7 (**P = 0.0024) (*n* = 3, and each replicate was seeded in 2 wells). Data were analyzed using unpaired Student’s *t* test, and multiple comparisons were corrected using Dunnett’s test. Magnification, 40×.

With proliferative and metastatic signatures enriched in the *DAB2IP*-low Luminal A breast tumors, we compared the effect of *DAB2IP* status on proliferation scores by molecular subtype. Proliferation scores (Supplemental Table 1) for the TCGA breast cancer samples are based on the 11-gene signature established by Ciriello et al. and Li et al. (10, 11). In the generally aggressive subtypes Basal-like, HER2-enriched, and Luminal B, we did not observe a significant difference in proliferation score by *DAB2IP* status. However, for Luminal A tumors, we observed a significantly higher proliferation score for *DAB2IP*-low tumors as compared with *DAB2IP*-high tumors (Figure 4A).

To test if loss of DAB2IP signaling contributes to tumor cell proliferation in Luminal A breast cancer cells, we performed DAB2IP knockdown in the Luminal A cell line T47D (25). Relative to controls, cell proliferation was significantly enhanced with knockdown of DAB2IP in T47D cells at both 24 hours and 48 hours after transfection (Figure 4B). Since the functional enrichment analysis with upregulated genes in low-*DAB2IP* Luminal A tumors displayed significant enrichment terms associated with cell cycle phase transition (Figure 2C) and other proliferative gene sets (Figure 2D), we then analyzed the effect of loss of DAB2IP on the expression of established proliferation and cell cycle genes in the Luminal A T47D cell line. We found that knockdown of DAB2IP elicited increased expression of a number of cell cycle and proliferation genes (Supplemental Figure 3A). Although we did not observe a difference in proliferation score based on *DAB2IP* levels for Luminal B tumors, when we silenced DAB2IP in Luminal B BT474 cells, we observed increased proliferation at the 24-hour time point (Supplemental Figure 3B). Additionally, a wound-healing assay showed that migration was significantly increased with knockdown of DAB2IP in T47D cells (Figure 4C).

Next, we performed a 3D multicellular tumor spheroid assay, which mimics the architecture of solid tumors (26). These multicellular spheroids are characterized by a necrotic core surrounded by a viable rim of quiescent cells and a peripheral layer of proliferating tumor cells (26). For this assay, we utilized T47D cells expressing scrambled shRNA or 2 independent shRNAs for DAB2IP (Figure 4D). Using an increasing number of cells, we observed that DAB2IP knockdown resulted in significantly larger spheroids by 48 hours (Figure 4E). As shown in Figure 4E, by day 4 of cell plating, DAB2IP knockdown T47D spheroid borders had lost uniformity, indicating proliferation at the periphery of spheroids (26). By day 7 of cell plating, DAB2IP knockdown spheroids had lost organization and compactness in the cancer cells (26), thereby supporting the hypothesis that loss of DAB2IP promotes an aggressive tumorigenic phenotype.

### DAB2IP suppression promotes a cancer stem-like cell phenotype in ER^+^ luminal cell lines.

Despite the availability of hormone therapies to inhibit the growth of ER^+^ cancer cells, studies have shown that 25%–40% of patients with breast cancer with luminal subtypes exhibit metastatic recurrence (27). These relapses have been associated with treatment-resistant TICs/cancer stem cells (CSCs) and activation of factors that contribute to CSCs (28). In the gene enrichment analysis, we observed that the *DAB2IP*-low Luminal A breast cancer subtype is associated with established CSC gene signatures (Figure 2F and Supplemental Table 2). Furthermore, in *DAB2IP*-low Luminal A tumors, we identified increased expression of individual breast cancer stemness-governing genes such as *EXOSC9* and *SKA3* (29, 30) (Figure 2A and Supplemental Figure 1B). Furthermore, knockdown of DAB2IP in T47D cells resulted in an increased expression of *BUB1* (Supplemental Figure 3A), which has been associated with the maintenance of cancer cell stemness (31). Thus, to address the potential that loss of DAB2IP promotes stemness, we performed tumorsphere assays using shDAB2IP and shControl in Luminal A T47D and Luminal B BT474 cell lines. Notably, knockdown of DAB2IP significantly increased primary tumorsphere formation in T47D cells, as shown in Figure 4F. Similarly, DAB2IP ablation in BT474 cells (Supplemental Figure 3C) resulted in a significant increase in tumorsphere formation associated with larger spheres (Supplemental Figure 3D). Thus, these results indicate that loss of DAB2IP favors an environment for CSC formation and maintenance in ER^+^ luminal breast cancer cells, which may underlie cancer aggressiveness.

### IKK/NF-κB pathway activation by loss of DAB2IP in the Luminal A breast cancer subtype.

**Figure 5:**
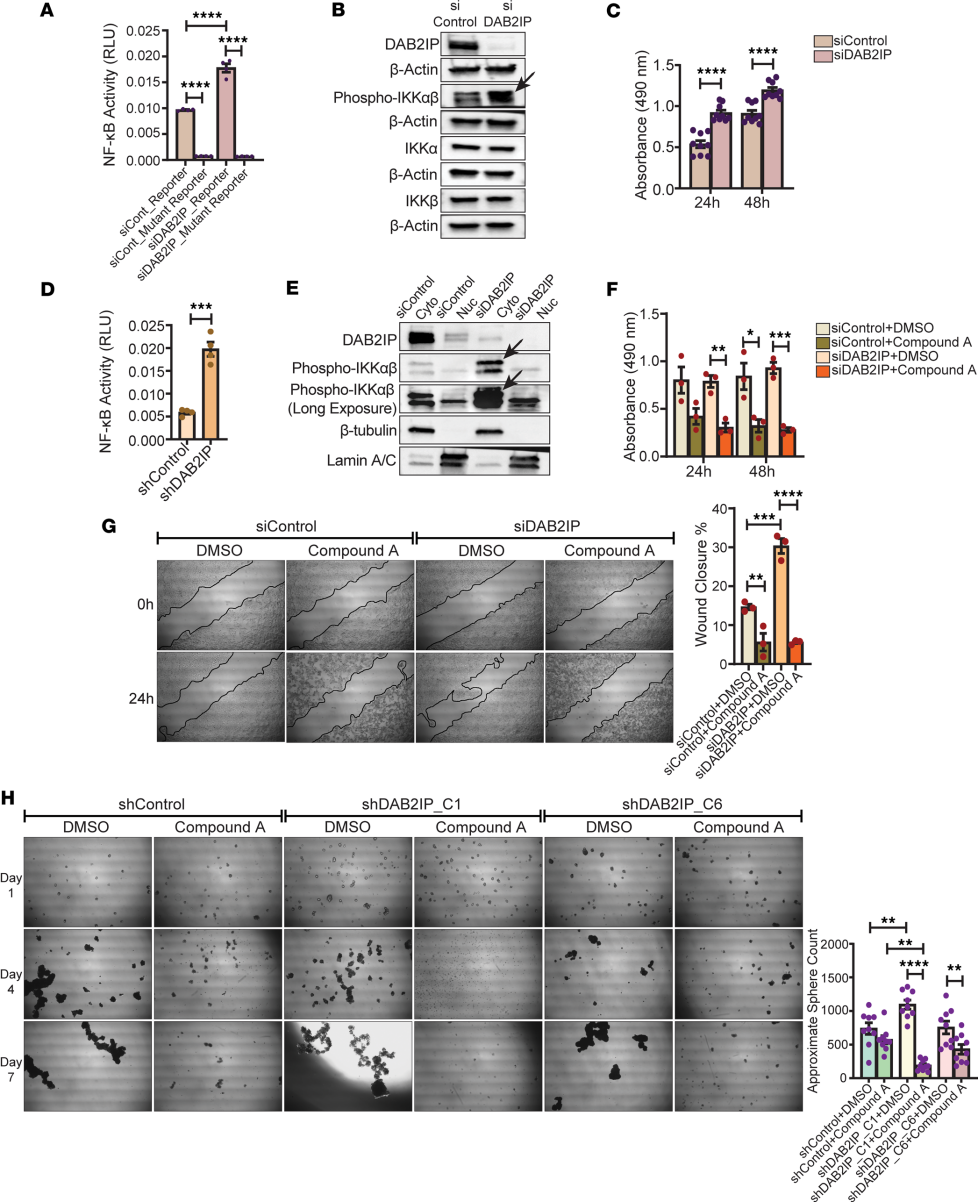
DAB2IP loss activates the IKK/NF-κB signaling pathway in Luminal A breast cancer cells. (**A**) After transfection with DAB2IP or control siRNA, MCF10A cells were transfected with WT or mutant 3×κB luciferase reporter plasmids and pRL-TK Renilla plasmid. Cell incubation for 24 hours was followed by dual luciferase assay (****P < 0.0001) (*n* = 4). (**B**) Immunoblot showed increased phospho-IKKαβ expression (arrow) in siDAB2IP MCF10A cells (*n* = 3). (**C**) After transfection, siDAB2IP and siControl MCF10A cell proliferation was determined by MTS assay (****P < 0.0001) (*n* = 9). (**D**) shDAB2IP and shControl T47D cells were transfected with WT or mutant 3×κB luciferase reporter constructs and pRL-TK Renilla construct for dual luciferase assay (***P = 0.0001) (*n* = 4). (**E**) Cytoplasmic and nuclear extracts from siDAB2IP and siControl T47D cells were used for immunoblotting to show an increase in the cytoplasmic phospho-IKKαβ levels (arrows) in siDAB2IP cells (*n* = 3). (**F**) Transfected T47D cells were treated with 5 μM compound A or DMSO every 8 hours, followed by MTS assay at 24 and 48 hours to assess proliferation (*P = 0.0282, **P = 0.0034, ***P = 0.0005) (*n* = 3). (**G**) After treatment with 5 μM compound A or DMSO, siDAB2IP and siControl T47D cells were subjected to scratch-wound assays (**P = 0.008, ***P = 0.0002, ****P < 0.0001) (*n* = 3). (**H**) shDAB2IP and shControl T47D cells were treated with 5 μM compound A or DMSO for tumorsphere assay, with images taken on days 1, 4, and 7 (**P = 0.004, **P = 0.0063, **P = 0.0018 ****P < 0.0001) (*n* = 3, seeded in 3 wells per replicate). Unpaired Student’s *t* test and multiple comparisons corrected with Dunnett’s test were used to analyze the data. Magnification, 40×.

As shown in Figure 2D, low *DAB2IP* in Luminal A tumors upregulated putative targets of the translation factor EIF4E, a reported transcriptional target of NF-κB (32). In addition, GO analysis of the METABRIC dataset showed that the upregulated genes in the *DAB2IP*-low ER^+^ group are associated with an enrichment of NF-κB signaling pathway genes (Supplemental Figure 1F and Supplemental Table 2). Furthermore, Olsen et al. showed that loss of DAB2IP in MCF10A cells resulted in enhanced NF-κB reporter activity (7). Taken together, these observations led us to investigate the association between the NF-κB signaling pathway and the loss of DAB2IP in the Luminal A breast cancer subtype. We first examined the effect of DAB2IP loss on the nonmalignant breast epithelial cell line MCF10A by utilizing WT or mutant NF-κB reporter genes. In these cells, DAB2IP knockdown significantly increased the activity of the WT NF-κB–dependent reporter compared with the control cells (Figure 5A), a finding consistent with the previous report (7). However, no such increase was observed in the siDAB2IP cells containing the mutant NF-κB reporter (Figure 5A). We also show that DAB2IP loss in MCF10A cells is associated with an increase in phosphorylation of IKKα/β, the upstream regulator of NF-κB (Figure 5B). In addition, we observed that DAB2IP knockdown significantly increased MCF10A cell proliferation at both 24 and 48 hours (Figure 5C). This was also reflected in an MCF10A RNA-Seq assay (Supplemental Figure 4A), wherein we found that the upregulated DEGs with knockdown of *DAB2IP* were enriched in oncogenic terms, including positive regulation of cell migration and positive regulation of cell motility, along with other protumorigenic biological processes (Supplemental Figure 4, B and C). Furthermore, we observed that one of the pathways that was enriched in *DAB2IP* knockdown MCF10A cells was the NF-κB signaling pathway (Supplemental Figure 4C).

To test the hypothesis that DAB2IP regulates NF-κB activity in Luminal A breast cancer cells, shControl and shDAB2IP T47D cells were transfected with an NF-κB–dependent reporter and Renilla plasmid. The results of the reporter assays demonstrate that NF-κB activity is significantly increased with DAB2IP loss in these cells (Figure 5D). Luminal B BT474 cells also exhibited increased NF-κB activity following DAP2IP knockdown (Supplemental Figure 4D), consistent with results from Olsen et al. (7). To address a mechanism of endogenous NF-κB regulation by loss of DAB2IP, T47D cells were transfected with control siRNA or with siRNA for DAB2IP, and we found that phosphorylation of IKKα/β, kinases that promote NF-κB activity, was increased in the cytoplasmic fraction of DAB2IP knockdown cell extracts (Figure 5E). Furthermore, we analyzed p65 (RELA) protein expression and association with low DAB2IP and corresponding clinical grade by performing IHC staining on the same ER^+^ breast cancer TMAs as in Figure 3, A–C. As stated before, these TMAs were comprised of 116 broadly ER^+^ tumors and NATs (Supplemental Table 4). Although we did not observe an increase in tumor nuclear localization of p65 with low DAB2IP, there was distinct stromal p65 cellular expression in patients with low DAB2IP displaying higher grades (Supplemental Figure 5, A and B), potentially related to an altered tumor microenvironment for these tumors.

Since DAP2IP loss resulted in increased phospho-IKKαβ levels (Figure 5E) and enhanced cell proliferation (Figure 4B), we hypothesized that blocking IKK would reverse the effect of DAB2IP knockdown, thereby decreasing cell proliferation. We sought to test this hypothesis by treating the DAB2IP knockdown and control T47D cells with either DMSO or compound A, a well-established IKK inhibitor (33), and then measured cell proliferation at 24 and 48 hours. At 24 hours after treatment, DAB2IP knockdown cells exhibited a significant reduction in proliferation in response to compound A compared with control (Figure 5F). At 48 hours, both DAB2IP knockdown and control T47D cells exhibited a significant decrease in proliferation rate when treated with compound A (Figure 5F). Next, we performed wound-healing assays on either DMSO or compound A–treated DAB2IP knockdown and control T47D cells and found that, at 24 hours, the migration rate was significantly disrupted in both compound A–treated DAB2IP knockdown cells and control T47D cells (Figure 5G). This was not unexpected, as IKK is active in T47D, presumably controlling proliferation and migration. Nevertheless, the effect of compound A on the migration and proliferation rate of DAB2IP-knockdown T47D cells at 24 hours and 48 hours, respectively, was more significant compared with that of the control cells (Figure 5, F and G). In addition, tumorsphere assays showed that sphere formation was significantly decreased in compound A–treated DAB2IP knockdown T47D cells compared with control cells (Figure 5H). These results suggest that loss of DAB2IP in Luminal A cancer cells enhances IKK activity to promote NF-κB signaling or other oncogenic-related pathways, which in turn contributes to cancer aggressiveness.

Since DAB2IP regulates the RAS signaling pathway (7), we then investigated the effect of loss of DAB2IP on the activation of p38 MAPK, a downstream effector of RAS (34). We found that knockdown of DAB2IP increased the levels of phosphorylated p38 in T47D cells (Supplemental Figure 5E). Furthermore, knockdown of p38 restored the migration rate of T47D cells, which was otherwise significantly increased by DAB2IP knockdown (Supplemental Figure 5F). We hypothesize that p38 contributes to certain low-DAB2IP oncogenic phenotypes in ER^+^ breast cancers.

### Identification of candidate NF-κB target genes in DAB2IP-low Luminal A breast tumors.

**Figure 6:**
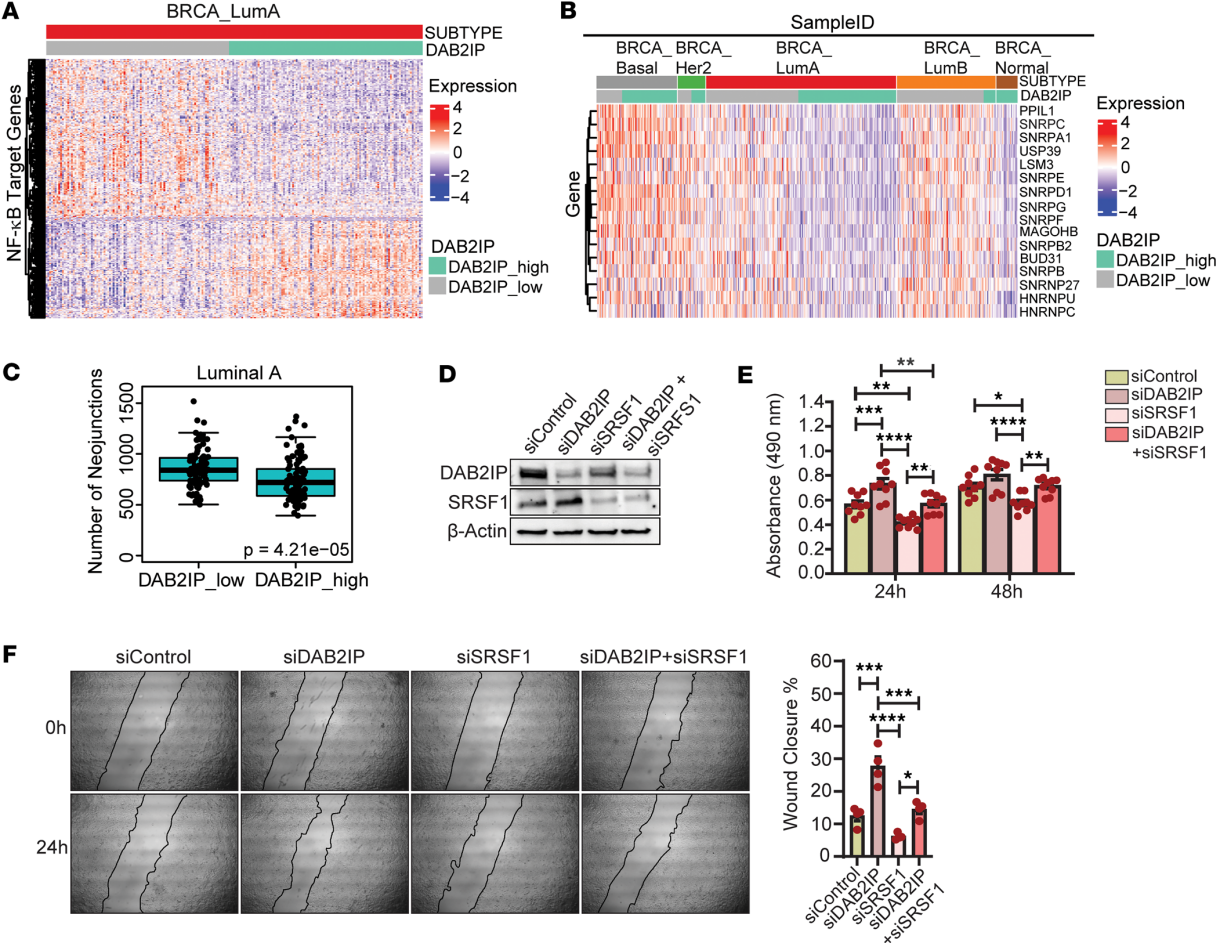
Effect of low *DAB2IP* on NF-κB target genes in Luminal A breast tumors. (**A**) Publicly available breast cancer RelA ChIP-Seq dataset was mined and analyzed to map putative NF-κB target genes based on high/low *DAB2IP* in the TCGA Luminal A subtype. (**B**) Heatmap displays 16 KEGG “spliceosome” pathway genes in Luminal A subtype based on *DAB2IP* levels, clustered across all breast cancer subtypes. (**C**) Neojunctions from TCGA breast cancer data were graphed based on high/low *DAB2IP* expression in Luminal A tumors (*P* = 4.21 × 10^–5^). (**D** and **E**) T47D cells transfected with siRNA against SRSF1, DAB2IP, or control were analyzed by immunoblotting and MTS assay. (24hrs: siControl vs siSRSF1, **P = 0.0033; siDAB2IP vs. siDAB2IP+siSRSF1, **P = 0.0014; siSRSF1 vs siDAB2IP+siSRSF1, **P = 0.0025; siControl vs siDAB2IP, ***P = 0.0008; siDAB2IP vs siSRSF1, ****P < 0.0001; 48hrs: siControl vs siSRSF, *P = 0.0112; siSRSF1 vs siDAB2IP+siSRSF1, **P = 0.0069; siDAB2IP vs siSRSF1, ****P < 0.0001) (*n* = 9). (**F**) After 24 hours of transfection, siControl, siDAB2IP, and/or siSRSF1 T47D cells were subjected to scratch-wound assay in 6-well plates (*P = 0.0144, ***P = 0.0001, ***P = 0.0004, ****P < 0.0001) (*n* = 4). Data were analyzed using unpaired Student’s *t* test, and multiple comparisons were corrected using Dunnett’s test.

NF-κB activity is associated with several oncogenic phenotypes, including effects on cell proliferation and survival, as well as promotion of the CSC phenotype and therapy resistance (35). We used the DEGs between high/low *DAB2IP* Luminal A tumors identified above to study the expression landscape of potential NF-κB target genes. Putative NF-κB target genes were grouped based on publicly available RelA ChIP-Seq data in breast cells (36). Signals within 5 kb of the transcription start site (TSS) of target genes were averaged for HMEC, MCF7, and MDA-MB-231 cells (*n* = 20 studies) and filtered to retain promoters with a signal greater than or equal to 10. We found that 253 of the proposed NF-κB target genes were differentially expressed based on *DAB2IP* levels in Luminal A breast tumors (Figure 6A and Supplemental Table 5). We identified candidate NF-κB targets such as BIRC5, TBCA, SRSF1, CDK5, and others involved in RNA splicing, cell proliferation, and endocrine resistance and found that they were significantly increased in the *DAB2IP*-low Luminal A tumors (Supplemental Figure 6A). Furthermore, several upregulated NF-κB target genes identified in the *DAB2IP*-low Luminal A group have been previously associated with cancer and specifically with breast cancer, such as: (a) the transcription factor ZNF652, which has been identified as a predictor of aggressive breast cancer (37); (b) Fragile X–related protein (FXR1), which promotes c-myc translation and is highly expressed in ovarian cancer (38); (c) NEDD8, which is a sumolyating enzyme that regulates NF-κB activity and is expressed in ER^+^ breast cancer, which correlates with a poorer prognosis (39); and (d) HNRNPA2B1, an RNA binding protein that activates the NF-κB pathway and promotes tumorigenesis (40). These results suggest the importance of NF-κB activity in *DAB2IP*-low Luminal A cells and tumors.

We then subjected the differentially expressed proposed NF-κB target genes in *DAB2IP*-low Luminal A tumors from Figure 6A to functional enrichment analysis. Interestingly, the upregulated putative NF-κB targets were highly enriched for RNA processing and splicing functions (Supplemental Figure 6B and Supplemental Table 2). Alternative splicing is common in cancer and often leads to altered expression of genes encoding proteins associated with the splicing machinery (41). We then examined the expression of genes involved in splicing kinetics (42, 43) and found that in the “KEGG spliceosome” pathway, 16 of the 145 genes were upregulated in *DAB2IP*-low Luminal A tumors (Figure 6B). Furthermore, these 16 genes were also upregulated broadly in Luminal B and Basal-like subtypes (Figure 6B). With this increase in expression of splicing factors, we examined if there was a change in neojunctions (novel cancer-specific exon-exon junctions) in different ER^+^ breast tumor datasets that was not observed in the normal-like tumor subset. Using a list of neojunctions generated from TCGA breast cancer data published by Kahles et al. (44), we found that Luminal B samples overall had a higher median neojunction load (median 876) than Luminal A (Supplemental Figure 6C and Supplemental Table 6). However, in Luminal A tumors, samples with low *DAP2IP* status had a higher median level of neojunctions (median 839) than tumors with higher expression of DAP2IP (Figure 6C). In Luminal B tumors, *DAP2IP* expression did not alter neojunction levels (Supplemental Figure 6D). The neojunction load in Luminal A low *DAP2IP* samples was similar to that observed in Luminal B samples with any *DAP2IP* expression level (*P* = 0.258, 2-tailed Student’s *t* test). Taken together, these results further suggest that loss of *DAB2IP* among Luminal A tumors is associated with a more aggressive Luminal B–like phenotype.

Consistent with the genomic results wherein *DAB2IP*-low Luminal A tumors had an upregulation of splicing factors (Figure 6B), we found that knockdown of DAB2IP in T47D cells resulted in increased expression of *SRSF1*, *HNRNPA2B1*, and *HNRNPU* RNA levels (Supplemental Figure 6E). We then determined whether inhibiting IKK would downregulate the expression of these proposed NF-κB–regulated splicing genes in *DAB2IP*-low Luminal A cells. Consistent with this hypothesis, DAB2IP knockdown cells, when treated with the IKK inhibitor compound A, exhibited decreased expression of the splicing genes *SRSF1*, *HNRNPA2B1*, and *HNRNPU* (Supplemental Figure 6E). This suggests that loss of DAB2IP expression plays a key role in regulating NF-κB targets, contributing to altered splicing in Luminal A breast cancer.

In addition, we scanned for binding motifs of NF-κB subunits in the DEGs between Luminal A low/high *DAB2IP* tumors and found that 129 upregulated genes in the *DAB2IP*-low Luminal A group contained binding motifs for NF-κB subunits p65 and p50 (Supplemental Figure 7A and Supplemental Table 7). We also found that some of these genes contained binding motifs for the NF-κB subunit c-Rel (Supplemental Figure 7A and Supplemental Table 7). We also noted that 32 of those 129 genes overlapped with the NF-κB candidate gene sets upregulated in the DAB2IP-low Luminal A subset (Supplemental Figure 7B).

Next, to investigate the effect of loss of DAB2IP on NF-κB target genes, we selected SRSF1, and BIRC5, which are known to play important protumorigenic roles in breast cancer (Supplemental Tables 3 and 5). Analyzing SRSF1 and BIRC5 protein expression on the same ER^+^ TMAs as in Figure 3, A–C, we found that with grade 2 and 3 tumors exhibiting loss of DAB2IP, there was a significant increase in SRSF1 expression (Supplemental Figure 5, A and C). We also observed an increase in BIRC5 protein expression, specifically in grade 3 tumors that displayed loss of DAB2IP (Supplemental Figure 5, A and D). Furthermore, we observed that, even though not significant, knockdown of DAB2IP increased SRSF1 expression at the protein level in T47D cells (Figure 6D). However, in these cells, we did not observe an effect of DAB2IP levels on BIRC5 expression (Supplemental Figure 8A). While our TCGA RNA-Seq analysis identified BIRC5 as one of the genes upregulated in Luminal A *DAB2IP*-low tumors, our cell-based ChIP-Seq analysis showed BIRC5 to be bound by p65 at the genomic level in control T47D cells (Supplemental Figure 8B). This observation is not surprising, as we have shown that IKK is active basally in T47D cells (Figure 5E).

We hypothesized that knockdown of SRSF1 or BIRC5 along with knockdown of DAB2IP would reverse the effect of loss of DAB2IP on the proliferation and migration rate of T47D cells. We found that the significant increase in proliferation and migration rates of T47D cells caused by the knockdown of DAB2IP was rescued by the knockdown of SRSF1 (Figure 6, E and F). Even though not significant, BIRC5 knockdown also showed a slight reversal of the increase in proliferation caused by the loss of DAB2IP at 24 and 48 hours (Supplemental Figure 8C). These results suggest that loss of DAB2IP in Luminal A breast cancer leads to increased NF-κB signaling and an increase in the expression of genes that are proproliferative and protumorigenic, thereby making this subset of Luminal A tumors more aggressive.

### Loss of DAB2IP enhanced both canonical and noncanonical NF-κB signaling.

**Figure 7:**
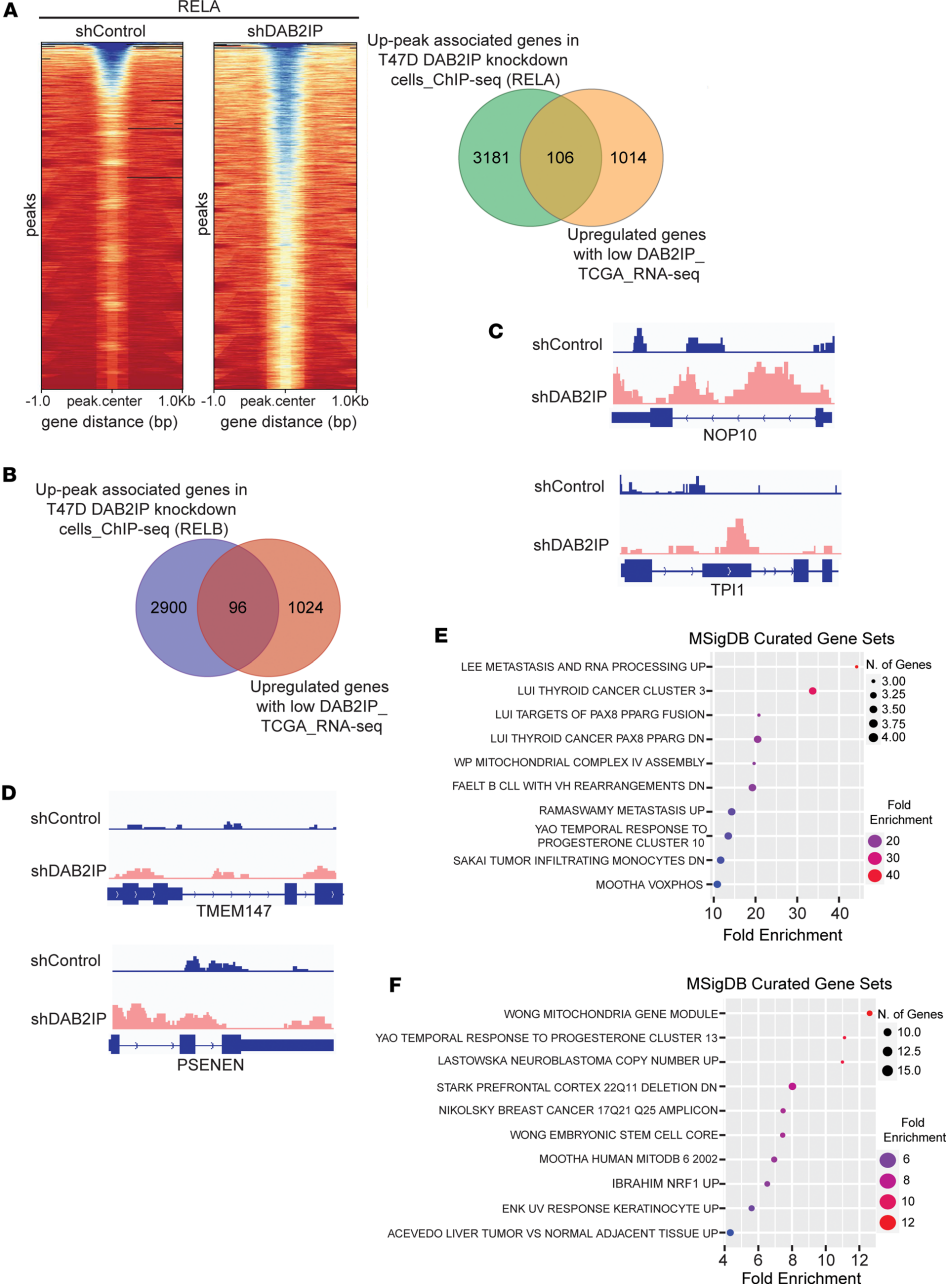
The effect of low DAB2IP on the genomic binding of NF-κB **subunits.** (**A**) Profile heatmaps around ± 1 kb of RefSeq gene TSS were created, displaying peak count levels with a color gradient (blue-to-red: high-to-low). The Venn diagram shows the overlap between up-peak RELA binding genes in shDAB2IP T47D cells and upregulated genes in the *DAB2IP*-low Luminal A TCGA dataset. (**B**) Venn diagram shows the overlap of up-peak RELB genes in shDAB2IP T47D cells and upregulated genes in the Luminal A *DAB2IP*-low TCGA group. (**C** and **D**) ChIP-Seq signal tracks were generated for *NOP10*, *TPI1*, *TMEM147*, and *PSENEN* using Integrated Genome Viewer software. (**E** and **F**) Common genes between TCGA RNA-Seq and RelA/RelB ChIP-Seq were processed for enrichment analysis using curated MSigDB gene sets (FDR > 0.05).

We determined the effect of NF-κB activation in *DAB2IP*-low Luminal A breast cancer by profiling RELA, RELB, and NFKB2 genomic binding and gene expression using ChIP-Seq in stable DAB2IP knockdown and control T47D cells. We found that, with DAB2IP knockdown, there were 4,735; 4,302; and 3,405 unique upregulated peaks associated with RELA/p65, RELB, and NFKB2 (Figure 7A and Supplemental Figure 9, A and B). Integrating the T47D cell–based ChIP-Seq and TCGA RNA-Seq analyses, we found that DAB2IP loss identified 106 RELA-regulated genes (Figure 7A) and 96 RELB-regulated genes (Figure 7B), respectively. For instance, genes like *NOP10* and *TPI1* exhibited increased RELA binding in *DAB2IP* knockdown cells and were correspondingly upregulated in the *DAB2IP*-low TCGA RNA-Seq gene set (Figure 7C). Similarly, RELB binding to *TMEM147* and *PSENEN* was enriched in DAB2IP knockdown cells as compared with control cells, which also exhibited increased expression in the *DAB2IP*-low TCGA RNA-Seq set (Figure 7D). Furthermore, GSEA GO analysis of these overlapped RELA and RELB binding genes identified the top enriched terms as associated with metastasis, proliferation, stemness, and cancer relapse (Figure 7, E and F). We also identified 71 genes bound by NFKB2 that were upregulated with low DAB2IP in both the ChIP-Seq and TCGA RNA-Seq datasets, which were enriched in terms associated with breast cancer (Supplemental Figure 9C). Thus, these results indicate that loss of DAB2IP in Luminal A ER^+^ breast cancer positively affects both canonical and noncanonical NF-κB signaling arms, which we propose contributes to an aggressive cancer-associated phenotype. Notably, comparison of genes between the DAB2IP-low T47D–based ChIP-Seq and *DAB2IP*-low Luminal A RNA-Seq data did not identify the NF-κB candidate genes such as *BIRC5*, *TBCA*, *SRSF1*, and *CDK5*. This may be explained by the ChIP-Seq results showing that some of these genes are bound basally by p65 in control T47D cells (Supplemental Figure 8B). Nevertheless, a significant number of proposed NF-κB target genes overlap between the ChIP-Seq and TCGA RNA-Seq datasets.

## Discussion

The loss of expression or activity of tumor suppressor proteins, including DAB2IP is a common theme across cancers (45). For breast cancer, research on DAB2IP loss is largely limited to the work of Cichowski and colleagues (7) who studied its loss within the Luminal B TCGA cohort. They found that low expression of *DAB2IP* occurs in approximately 50% of Luminal B cancers and is associated with poor outcomes. We show here that approximately 25%–30% of Luminal A breast cancers exhibit low *DAB2IP* expression and that this is associated with poor relapse-free survival and a significantly higher ROR score (Figure 1D). This indicates that the clinical significance of low *DAB2IP* is not just limited to Luminal B cancer but also to the more common Luminal A subtype.

Similar to reports regarding genetic changes that promote more aggressive ER^+^ breast cancer phenotypes (46–49), we found that the differential gene expression pattern in Luminal A *DAB2IP-*low tumors correlates closely with genes expressed in Luminal B and HER2 tumors, irrespective of their *DAB2IP* status (Figure 2, A and B, and Supplemental Figure 1B). Pathway analysis revealed that the upregulated genes in *DAB2IP*-low Luminal A tumors were associated with cell proliferation, EMT, stemness, and ER signaling/endocrine therapy resistance (Figure 2, C–F). Furthermore, we demonstrated that *DAB2IP*-low Luminal A tumors exhibited more shallow genomic deletions compared with the *DAB2IP*-high group (Figure 1E), also indicating that loss of DAB2IP expression was associated with more aggressive cancer phenotypes.

DAB2IP is inactivated in cancers through promoter methylation involving the EZH2-PRC2 complex, phosphorylation by AKT1, posttranscriptional silencing by microRNAs, and degradation by E3-ubiquitin ligases (24). Here we show that DAB2IP protein expression is lost in some grade 2/stage II or higher ER^+^ breast tumors, thereby suggesting that DAB2IP loss in ER^+^ breast cancer contributes to a more aggressive stage of disease (Figure 3). Further experimentation is needed to determine the mechanisms whereby DAB2IP expression is reduced in breast cancer.

Consistent with gene enrichment/pathway analysis (Figure 2C), *DAB2IP*-low Luminal A tumors display a significant increase in proliferation score as compared with the *DAB2IP*-high group (Figure 4A). Notably, we did not detect a difference in the Luminal B proliferation score relative to *DAB2IP* expression, and this may be due to a lack of statistical power due to enrichment of proliferation markers as a criterion in subtype designation to Luminal B or that low DAB2IP does not promote proliferation over an existing high-proliferation background. Thus, the presumed oncogenic effect of low *DAB2IP* expression on the Luminal B subtype (7) might occur through another mechanism. Tumorsphere assays demonstrated that DAB2IP silencing indeed increased the relative number and sizes of spheres in vitro, more so in the Luminal B cell line (Figure 4F and Supplemental Figure 3D). Thus, one possibility is that, since Luminal B tumors are generally more proliferative, loss of DAB2IP promotes an aggressive state by promoting stemness, whereas DAB2IP loss in the Luminal A subtype leads to more aggressive characteristics ranging from proliferation to CSC-like features.

We found that knockdown of DAB2IP led to a significantly increased NF-κB reporter activity in the Luminal A cell line associated with an increase in phosphorylation of IKK, a critical upstream regulator of the NF-κB pathway (Figure 5, D and E). The NF-κB pathway is strongly linked with a variety of oncogenic mechanisms, including proliferation, stemness, metastasis, and endocrine therapy resistance (50–52). Using published RelA ChIP-Seq data from cell lines (36), we derived a proposed set of NF-κB target genes that exhibited significantly altered expression between *DAB2IP* high and low Luminal A groups (Figure 6A). Genes such as *BIRC5*, *SRSF1*, *CDK5*, and *TBCA* were highly expressed in Luminal A *DAB2IP*-low tumors compared with *DAB2IP*-high tumors. However, no such difference was observed in other subtypes (Supplemental Figure 6A). Furthermore, at the protein level, with loss of DAB2IP, we observed an increase in the expression of SRSF1 in T47D cells (Figure 6D) and in ER^+^ TMAs (Supplemental Figure 5, A and C). However, we did not observe a distinct difference in BIRC5 levels with loss of DAB2IP at the protein level in T47D cells (Supplemental Figure 8A). ChIP-Seq analysis indicates that BIRC5 was bound by p65 in control T47D cells (Supplemental Figure 8B). Thus, cultured T47D cells exhibited a basal level of NF-κB activation, which may have obscured the effects of DAB2IP knockdown on some gene targets.

Furthermore, we identified upregulation of NF-κB target genes associated with RNA splicing in the *DAB2IP*-low Luminal A subset (Supplemental Figure 6B). We found 16 spliceosome genes to be upregulated in *DAB2IP*-low Luminal A tumors (Figure 6B). While some of these spliceosome genes, such as *SNRPA1*, *SNRPD1*, *USP39*, *HNRNPU*, and *HNRNPC*, have been shown to play an important role in promoting TNBC cell survival, proliferation, and response to chemotherapy (53–57), others are yet to be studied in breast cancer. Interestingly, these genes were also upregulated in Basal-like and Luminal B subtypes irrespective of their *DAB2IP* status (Figure 6B). In addition, the increase in neojunction load in Luminal A *DAP2IP*-low tumors (Figure 6C) was similar to that observed in Luminal B samples with any *DAP2IP* expression level. These observations, in combination with the results of the cell-based studies, lead us to hypothesize that loss of *DAB2IP* in Luminal A tumors is associated with a more aggressive Luminal B– or HER2-enriched–like phenotype. Furthermore, inhibition of IKK signaling led to a decrease in the expression of *SRSF1*, *HNRNPA2B1*, and *HNRNPU*, consistent with regulation by NF-κB (Supplemental Figure 6E).

To directly test whether loss of DAB2IP promotes enhanced association of NF-κB subunits with genomic targets, we performed ChIP-Seq analysis and found that loss of DAB2IP in the Luminal A cell line T47D resulted in increased binding of both RELA and RELB across the genome as compared with control (Figure 7, A and B), indicating the activation of both canonical and noncanonical NF-κB. For instance, RELA-bound genes such as *NOP10*, a member of the H/ACA small nucleolar RNP (snoRNP) gene family, and *TPI1*, an important glycolytic enzyme are associated with poor prognosis in breast cancer (58, 59) (Figure 7C). Similarly, DAB2IP knockdown led to increased RELB binding to *TMEM147*, which is known to regulate cell proliferation, and *PSENEN*, a prognostic marker in low-grade gliomas (60, 61) (Figure 7D). Thus, we hypothesize that low DAB2IP in Luminal A tumors augments both canonical and noncanonical NF-κB signaling that subsequently results in increased binding of both RELA and RELB to genes that are associated with aggressiveness in this subset. This is also consistent with the increased phosphorylation of both IKKα and IKKβ with knockdown of DAB2IP (Figure 5E). Furthermore, IKK is known to phosphorylate substrates that are distinct from traditional NF-κB signaling; thus, these pathways may be relevant to DAB2IP-low luminal breast cancers (62, 63). Additionally, we found that loss of DAB2IP increases p38 phosphorylation, and p38 knockdown partly rescued the effect of DAB2IP knockdown in T47D cells (Supplemental Figure 5, E and F).

We also found that upregulated genes with low *DAB2IP* in Luminal A breast tumors were enriched in nonclassical estrogen and ESR1-regulated genes, which may affect responses to endocrine therapies and also drive proliferation (Figure 2E). Previously, Franco et al. found that inflammatory cytokine–induced NF-κB functions with ER to target and regulate genes not controlled by either transcription factor alone (64). Thus, NF-κB activated by loss of DAB2IP may lead to altered ER-controlled gene expression. Future studies exploring the functional association between ER and NF-κB signaling in low DAB2IP ER^+^ tumors are needed to provide insight into mechanisms whereby this tumor subset exhibits aggressive oncogenic phenotypes.

## Methods

### Sex as a biological variable.

Our study analyzed RNA-Seq datasets from female patients with breast cancer; therefore, sex was not considered as a biological variable in this study.

### Cell lines.

T47D (ATCC-HTB-133) and BT474 (ATCC-HTB-20) cell lines were cultured in RPMI-1640 medium (Invitrogen, 11875119) with 10% FBS (VWR, 97068-085) and 1% penicillin/streptomycin (Invitrogen, 15140-122). MCF10A cells (ATCC-CRL-10317) were cultured in DMEM/F12 medium (Invitrogen, 11330-032) supplemented with 5% horse serum (Invitrogen, 16050-122), 10 μg/mL recombinant human insulin (Invitrogen, 12585-014), 20 ng/mL recombinant epidermal growth factor (PeproTech, AF-100-15-100UG), 100 ng/mL cholera toxin (Sigma-Aldrich, C8052-2MG), 0.5 μg/mL hydrocortisone (Sigma-Aldrich, H0135-1MG), and 1% penicillin/streptomycin. Cells were maintained at 37°C with 5% CO_2_, validated by STR profiling, and routinely tested for mycoplasma contamination.

### Datasets and gene expression analysis.

TCGA breast tumor data corresponding to the Pan-Can Atlas (2018) release, including clinical annotations like hormone receptor status and tumor subtype, were retrieved from cBioPortal (8). The study utilized median-centered *Z* scores for all tumors, excluding those without subtype data. Differential expression analysis compared ER^+^ nonbasal and Luminal A–only tumors in the highest and lowest *DAB2IP* expression quartiles using DESeq2 (65). DAB2IP_mid are the cumulative samples in the second and third quartiles. Heatmaps and Pearson correlations were constructed using tidyHeatmap (66) and ComplexHeatmap (67). ROR-P scores and proliferation scores based on the 11-gene signature were provided for the TCGA breast cancer samples as performed by Ciriello et al. and Li et al. (10, 11), joined with other data based on tumor ID, and plotted using ggplot2 (68). Putative NF-κB target genes were defined based on publicly available RelA ChIP-Seq breast cancer data (ChIP-Atlas) (36). Average signal within 5 kb of the TSS of target genes was calculated for HMEC, MCF7, and MDA-MB-231 cells (*n* = 20 studies) and filtered to retain promoters with signal > 10. DEGs implicated in the KEGG splicesome pathway (42, 43) (https://www.genome.jp/dbget-bin/www_bget?pathway:hsa03040) were plotted using tidyHeatmap. Significance in ROR-P or proliferation scores between tumor groups was determined using Student’s *t* tests (*P* < 0.05).

*DAB2IP* expression levels in METABRIC were stratified into low (first quartile) and high (fourth quartile). The Samr package (69) detected DEGs in *DAB2IP*-low/high ER^+^ samples. Gene log_2_ expression associations with *DAB2IP* class were calculated using unpaired *t* tests, and significantly expressed genes were identified as those whose observed relative difference was greater than expected based on chance alone and that had a FDR corrected *P* < 1 × 10^–10^. DEGs were clustered by complete clustering and Euclidean distance and then annotated by *DAB2IP* expression class and PAM50 subtype.

### Survival and pathway analysis.

The Kaplan-Meier Plotter website (https://kmplot.com/analysis/) was used to generate the survival curves. For enrichment analysis, gene sets from MSigDB and relevant publications were screened for GO biological processes, oncogenic signatures, and keywords of interest pertaining to breast cancer, including estrogen signaling, endocrine therapy response, and stemness. DEGs in *DAB2IP*-low Luminal A tumors were then input to analyze set enrichment using the clusterProfiler R package. Gene ratios were computed as the number of genes enriched relative to the total number of genes from the specified set, plotted according to gene ratios and *q* values, and grouped by categories of interest. Overall trends per category were determined based on gene ratio predominance and the relative frequency of enriched gene sets. Enrichment analysis of genes overlapped between ChIP-Seq and RNA-Seq was performed using ShinyGO v.0.77 (70).

### IHC.

The breast cancer TMAs used in this study were purchased from TissueArray.Com LLC (TMA; BC081116e, BC081120g, BR1507). The slides were processed following a series of steps: deparaffinization with xylene, rehydration with decreasing ethanol concentrations, antigen retrieval in antigen unmasking solution (Vector Laboratories, H-3300-250), quenching endogenous peroxidase activity with Bloxall solution (Vector Laboratories, SP-6000-100), and blocking with blocking solution (10 mM Tris-HCl [Thermo Fisher Scientific, BP153-1]; 0.1M magnesium chloride [Sigma Aldrich, M8266-100G]; 0.5% Tween-20 [Thermo Fisher Scientific, BP337-500]; 1%BSA [Sigma Aldrich, A3059-100G]; and 10% goat serum [Vector Laboratories, S-1000-20]). Slides were then incubated with anti-DAB2IP antibody (Abcam, ab87811), anti-SRSF1 (Abcam, ab133689), anti-BIRC5 (Cell Signaling Technology [CST], 2808), or anti-P65 (CST, 8242) overnight in a humidified chamber at 4°C. Next, slides were treated with secondary antibody (Vector Laboratories, BA-1000), followed by incubation in Vectastain ABC solution (Vector Laboratories, PK-6100). Slides were incubated using the 3,3’-diaminobenzidine kit (Vector Laboratories, SK-4100), counterstained with hematoxylin, dehydrated, and mounted using mounting medium (Vector Laboratories, H-5000-60).

### Digital image analysis.

Image analysis at the UNC Lineberger Comprehensive Cancer Center Pathology Services core included using the Aperio AT2 digital scanner to capture images of stained TMA slides at 20× magnification. The DAB staining intensity was quantified by a boarded veterinary pathologist using Definiens Architect XD 64. The computer measured the average chromogen intensity of the cytoplasm and nucleus, which was validated qualitatively by a veterinary pathologist.

### siRNA transfections.

Cells were transfected 24 hours after plating at 70% confluence with siRNA (Dharmacon, M-008249-01-0010) using Lipofectamine 3000 (Thermo Fisher Scientific, L3000015) as per manufacturer’s instructions. Control cells were transfected with On-Target plus nontargeting control pool (Dharmacon, D-001810-10-20).

### shRNA transduction.

In total, 6 × 10^5^ cells in 6-well plates were transduced with DAB2IP-targeting lentiviral shRNAs or empty vector. Following 24-hour maintenance in regular medium, cells were subjected to puromycin (2 μg/mL) selection for 72 hours, and DAB2IP knockdown was validated by Western blot. The details of the DAB2IP shRNA clones used are as follows: shRNA#1: Lenti-shRNA Core Facility, University of North Carolina at Chapel Hill, Clone ID: TRCN0000001457; shRNA#2: DAB2IP MISSION shRNA Plasmid, Sigma Aldrich, Clone ID: TRCN0000414427.

### Proliferation and migration assays.

To determine the effect of loss of DAB2IP on T47D, BT474, and MCF10A cell proliferation, after 24 hours of siRNA transfection, 7,000 cells per well were seeded in 96-well plates. Proliferation assay was performed using the CellTiter 96 AQueous proliferation assay kit (Promega, G3580) following the manufacturer’s instructions at 24 and 48 hours after cell seeding.

For inhibition of the NF-κB pathway and subsequent proliferation assay, siDAB2IP and siControl cells were seeded in 96-well plates and treated at every 8-hour interval for 48 hours with either 5 μM of compound A or DMSO. A proliferation assay was performed at 24 and 48 hours of treatment using the CellTiter 96 AQueous proliferation assay kit following the manufacturer’s instructions.

To determine cell migration in DAB2IP knockdown or control cells, a scratch wound assay was performed (71). Compound A or DMSO-treated and/or untreated T47D siDAB2IP and siControl cells were starved overnight using Opti-MEM medium (Invitrogen, 31985-070), and a scratch was created in the cell monolayer (71). Images taken at 0 and 24 hours were analyzed using ImageJ software (NIH). Data were presented as the percentage by which the scratch area has decreased after 24 hours for each condition as compared with the scratch made at 0 hours.

### Tumorspheroid assay.

In total, 1,000; 2,000; and 4,000 DAB2IP stable knockdown or control T47D cells were seeded into Nunclon Sphera plates (Thermo Fisher Scientific, 174925) using RPMI-1640 medium, centrifuged at 250*g* for 5 minutes, and incubated at 37°C with 5% CO_2_ for 7 days. Spheroid images were taken on days 2, 4, and 7 with diameters measured using ImageJ software and graphed.

### Tumorsphere assay.

BT474 and T47D DAB2IP stable knockdown or control cells were collected and resuspended in 10 mL of MammoCult medium (Stemcell Technologies, 05620). Cells were centrifuged at 500*g* for 3 minutes, resuspended in 2 mL of MammoCult medium, and counted. In total, 5,000 BT474 and 3,000 T47D cells were plated in each well of 6-well ultra–low adherent plates and incubated at 37°C with 5% CO_2_ for 7 days. Images were taken at days 1, 4, and 7. Tumorspheres > 60 μM were enumerated on day 7.

### Dual luciferase assay.

WT or mutant 3×κB luciferase reporter constructs, containing 3 copies of the NF-κB binding site from the major histocompatibility complex class I gene positioned upstream of the luciferase reporter gene, and pRL-TK Renilla luciferase construct were transfected into DAB2IP-knockdown or control cells. After 24 hours of incubation, luciferase assay was performed using the dual luciferase assay kit (Promega, E1910) according to the manufacturer’s instructions, and results were normalized to Renilla luciferase activity.

### Nuclear/cytosolic fractionation.

To assess the nuclear/cytoplasmic Phospho-IKK-α/β expression, T47D DAB2IP knockdown or control cells were harvested, centrifuged (2,400*g* for 30 seconds), resuspended in 0.1% NP-40/PBS buffer supplemented with protease inhibitor (EMD Millipore, 11873580001) and phosphatase inhibitor (Sigma-Aldrich, P0044), and centrifuged at 2,000*g* for 2 minutes. The cytoplasmic fraction was collected for immunoblotting. The pellet was washed with 1 mL of 0.1% NP-40/PBS buffer and centrifuged for 2 minutes at 2,000*g*. Next, the pellet was lysed in RIPA buffer and sonicated for 10 minutes. The lysate was centrifuged at 17,000*g* for 10 minutes, and nuclear fraction was collected for immunoblotting.

### Immunoblotting.

DAB2IP knockdown and control cells were harvested, lysed with RIPA buffer, incubated on ice for 20 minutes, and centrifuged for 17,000*g* 10 minutes, and supernatant was collected for protein quantification using Bradford protein assay. Protein lysates were denatured at 97°C for 5 minutes, separated in 4%–15% Mini-Protean precast gels (Bio-Rad, 4561084), transferred to 0.2 μm nitrocellulose membrane using Trans-blot turbo transfer system (Bio-Rad), and blocked in 5% nonfat milk in 1× TBS/Tween-20 for 1 hour at room temperature. After primary antibody incubation overnight (anti-DAB2IP, Abcam, ab87811; anti–phospho-IKK-α/β, CST, 2697; anti-IKKα, CST, 2682; anti-IKKβ, CST, 8943; anti–β-Actin, CST, 3700; anti-SRF1, CST, 14902; anti-BIRC5, CST, 2808), membranes were incubated with secondary antibodies (Promega, W4011 and W4021) for 1 hour at room temperature and developed using Clarity Western ECL Substrate (Bio-Rad, 1705061).

### Quantitative PCR.

Total RNA was extracted using Quick-RNA Miniprep kit (Zymo Research, R1055) following the manufacturer’s protocol. cDNA synthesis was done using iScript kit (Bio-Rad, 170-8891). Probes were bought from Thermo Fisher Scientific. Fold change was calculated, and gene expression was quantified relative to GAPDH mRNA.

### RNA-Seq and analysis.

Total RNA from MCF10A DAB2IP knockdown and control cells was sent to Novogene Corporation Inc. for library preparation and RNA-Seq on Illumina NovaSeq 6000 (PE150) platform. According to Novogene’s overview of services, raw data were processed for quality control, aligned, and mapped to the reference genome, and Fragments Per Kilobase of transcript sequence per Millions base pairs sequenced (FPKM) was used to estimate gene expression levels.

### ChIP.

ChIP was performed as previously described (72). Briefly, 10 million stable DAB2IP knockdown or control T47D cells were cross-linked, lysed, and sonicated. For each immunoprecipitation, 25 μg DNA was incubated with primary antibodies (anti-p65, CST, 8242; anti-RelB, CST, 4922; anti-NFKB2, CST, 4882) for 1 hour and incubated with blocked protein G beads for 40 minutes at 4°C. After washing, reverse cross-linking was done, and DNA was purified using Qiagen PCR purification kit.

### ChIP-Seq.

ChIP samples and corresponding inputs were sent to Novogene Corporation Inc. for library preparation and deep sequencing using the NovaSeq (PE150) platform. According to Novogene’s documentation, sequences were mapped to the reference genome using Burrows Wheeler Alignment tool v 0.7.12, and peak calling was done with MACS2 v 2.1.0 (Supplemental Table 7).

### Statistics.

Statistical analysis and graphing were done using GraphPad Prism v.9.0. Data are shown as mean ± SEM and analyzed using unpaired Student’s *t* test, and multiple comparisons were corrected using Dunnett’s test, with replicate numbers provided in figure legends.

### Data availability.

The ChIP-Seq and RNA-Seq data are available at the NCBI Gene Expression Omnibus database (accession GSE227877). The R scripts used for data analysis and visualization in this study have been uploaded to GitHub (https://doi.org/10.5281/zenodo.14047811). Other datasets are available in the Supporting Data Values XLS file.

## Author contributions

ASB conceived the project. ASB, JMS, DPH, AM, PMS, and MAT designed the project. JMS analyzed TCGA data. AM performed experiments and interpreted data. ALE validated ChIP DNA fragment size. SVA and MAT analyzed METABRIC data. AM and RTK performed GSEA. PMS provided clinical interpretation of the patient’s tissue staining data. TL and KAH analyzed data related to TCGA neojunctions. Manuscript written by ASB, AM, and JMS with inputs from all of the other authors.

## Supplementary Material

Supplemental data

Unedited blot and gel images

Supplemental table 1

Supplemental table 2

Supplemental table 3

Supplemental table 4

Supplemental table 5

Supplemental table 6

Supplemental table 7

Supporting data values

## Version 1 10/17/2024

In-Press Preview

## Version 2 12/06/2024

Electronic publication
