# Supplementary material for: DAB2IP loss in luminal a breast cancer leads to NF-κB–associated aggressive oncogenic phenotypes
Source: JCI Insight. 2024 Dec 6;9(23):e171705. doi: 10.1172/jci.insight.171705 (PMC11623953; doi:10.1172/jci.insight.171705)
Supplement: Supplemental data [file jciinsight-9-171705-s051.pdf]

Supplementary Information

Supplementary Figure S1. ***DAB2IP*** **expression across breast cancer subtypes in ER+ TCGA and METABRIC cohorts. (A)** METABRIC breast cancer microarray gene expression matrix was downloaded to analyze *DAB2IP* expression across breast cancer molecular subtypes. **(B)** DESeq analysis of TCGA non-basal ER+ breast cancer RNA-seq data was performed, and DEGs were clustered across all breast cancer subtypes. Quartile-based cutoff allowed division of patients into high/low *DAB2IP*. The significance was determined at adjusted p<1e-10. **(C)** Down-regulated DEGs in *DAB2IP*-low TCGA Luminal A samples were analyzed for gene enrichment using GO:Biological Processes (BP) signatures (FDR cutoff >0.05). **(D)** METABRIC breast cancer gene expression map shows clusters of ER+ DEGs based on high/low *DAB2IP* (quartile-based cutoff) across breast cancer subtypes**. (E) and (F)** ShinyGO platform was used to perform gene enrichment analysis using MSigDB oncogenic pathway activation gene sets and Hallmark gene sets, respectively, for upregulated DEGs in *DAB2IP*-low ER+ METABRIC samples (FDR cutoff >0.05). Statistical analysis were performed using Student's t-tests.

Supplementary Figure S2. Higher grade tumors are associated with increased Ki67 Index percentage, and additional representative images showed that some high grade tumors retained DAB2IP expression. (A) TMAs consisting of 116 ER+ patients were stained for DAB2IP. **(B)** Ki67 index % value available for 89 of the 116 ER+ tumors was graphed against tumor grades. **(C)** Additional images of Luminal A TMA consisting of 126 patients showing DAB2IP expression. Data was analyzed using Student's t-tests.

Supplementary Figure S3. Effect of loss of DAB2IP on proliferation and tumorsphere formation in Luminal A and Luminal B cell lines. (A) Cell cycle and proliferation genes showed increased expression in DAB2IP knockdown Luminal A T47D cells compared to control cells as determined by quantitative RT-PCR (n=3). **(B)** Luminal B BT474 cells, after 24 hour transfection, were plated for MTS assay, and absorbance was measured after 24 and 48 hours (*p=0.0139) (n=9). **(C)** Control and DAB2IP-trageting shRNAs were used to transduce BT474 cells, and immunoblot was performed to determine the knockdown efficiency. **(D)** shDAB2IP and shControl BT474 cells were plated for tumorsphere assays, and images were acquired at Day 1, Day 4, and Day 7. (*p=0.0147, **p=0.0034) (n=3, and each biological replicate was seeded in 2 wells). Data was analyzed using Student's t-tests.

Supplementary Figure S4. Effect of loss of DAB2IP expression on the transcriptome of MCF10A cells. (A) MCF10A cells were transfected with siRNA against DAB2IP or non-targeting control pool, and RNA-sequencing was performed. DESeq analysis shows distinct clusters of DEGs between knockdown and control DAB2IP cells**. (B) and (C)** Gene enrichment analysis using GO: biological processes and MSigDB Hallmark gene sets were performed, respectively, using upregulated DEGs in DAB2IP knockdown MCF10A cells (FDR cutoff>0.05). **(D)** Post transfection with DAB2IP or control siRNA, BT474 cells were transfected with either wild-type or mutant 3×κB luciferase reporter constructs along with pRL-TK Renilla luciferase construct, and a dual luciferase assay was performed (n=4). Data was analyzed using Student's t-tests.

Supplementary Figure S5. Loss of DAB2IP increases P65, SRSF1, and BIRC5 expression in matched ER+ breast cancer tissues, and loss of p38 rescues migration of T47D cells. (A)

Matched representative images of DAB2IP, P65, SRSF1, and BIRC5 expression from 116 ER+ breast cancer specimens are shown. Arrows indicate positive cells. **(B)** Stromal P65 expression was quantified by computational measurements of staining intensity per specimen and plotted by tumor grades (***p=0.0002, ****p<0.0001). **(C)** SRSF1 **s**taining intensity ratio of average nucleus to average cytoplasm was plotted against tumor grades (*p =0.0177, **p=0.0033, ***p=0.0009). **(D)** Ratio of average nucleus to average cytoplasm of BIRC5 **s**taining intensity was graphed by tumor grades (***p=0.0008). **(E)** Immunoblot was performed using siDAB2IP and siControl T47D cells to show the increase in expression of phospho-p38 in DAB2IP knockdown cells (n=3). **(F)** Post 24-hour of transfection with siControl, siDAB2IP, and/or sip T47D cells, scratch-wound assay was performed (**p =0.0071, **p =0.0025, **p =0.0032, ***p =0.0001) (n=3). Data was analyzed using Student's t-tests.

Supplementary Figure S6. Impact of loss of DAB2IP on NF-κB target genes and neojunctions in Luminal A and Luminal B tumors and cell lines. (A) *DAB2IP*-low Luminal A subtype exhibited increased expression of NF-κB targets *BIRC5* adj.p=2.2115212243978402e-7), *CDK5* (adj.p=1.0657855824892208e-8), *SRSF1* (adj.p=1.6454635733683117e-11) and *TBCA* (adj.p=5.0683220299522466e-11). **(B)** Gene enrichment analysis using GO:BP was performed for upregulated NF-κB DEGs in Luminal A DAB2IP-low tumors (FDR cutoff>0.05). **(C)** Neojunction numbers mined from the TCGA breast cancer cohort were plotted by Luminal A and Luminal B subtypes (t-test p = 3.81e-10). **(D)** The number of neojunctions from the TCGA breast cancer cohort was plotted against high/low *DAB2IP* expression in Luminal B tumors. **(E)** siDAB2IP and siControl T47D cells were seeded in 96-well plates and treated with DMSO or 5 µM Compound A for 4 hours. Expression of NF-κB regulated splicing genes SRSF1, HNRBPA2B1, and HNRNPU at the mRNA levels was analyzed by quantitative RT-PCR (n=3). Data was analyzed using Student's t-tests.

Supplementary Figure S7. Several genes that were upregulated with low *DAB2IP* in Luminal A tumors are potentially regulated by the NF-κB signaling pathway. (A) NF-κB transcription factor was selected for motif search, and using the online software Motifmap, (http://motifmap.ics.uci.edu/), upregulated genes in *DAB2IP*-low Luminal A tumors were processed to identify genes containing binding sites of NF-κB subunits. **(B)** Venn diagram showing the overlap between the upregulated genes in the *DAB2IP*-low Luminal A subset containing NF-κB binding sites and the NF-κB candidate gene sets that we analyzed using publicly available RelA ChIP-seq data.

Supplementary Figure S8. Effect of loss of DAB2IP on NF-κB target gene BIRC5 in T47D cells. (A) T47D cells transfected with siRNA specific to BIRC5, DAB2IP, or control were analyzed by immunoblotting. **(B)** IGV platform was used to analyze the ChIP-seq signal tracks for BIRC5. (C) Post transfection with siControl, siDAB2IP, and/or siBIRC5, T47D cells were subjected to MTS assay (BIRC5: 24hrs-*p=0.0181, *p=0.0270, *p=0.0120; 48hrs-*p=0.0286) (n=9). Data was analyzed using Student's t-tests.

Supplementary Figure S9. ChIP-seq reveals differential NF-κB binding in DAB2IP knockdown Luminal A cell line. (A) Profile heatmap around TSS of RefSeq genes with read counts spanning a region of ± 1 kb around TSS are shown for RelB, and NFKB2. Blue-to-red color

gradient shows high-to-low counts in the corresponding region**.** **(B)** Venn diagram showing overlap between up-peaks between shControl and shDAB2IP T47D cells for RELA, RELB and NFKB2 binding. **(C)** Venn diagrams showing the overlap of up-peak associated NFKB2 binding genes in DAB2IP knockdown T47D cells and upregulated genes in low *DAB2IP* Luminal A TCGA analyzed dataset. Common genes between TCGA RNA-seq and NFKB2 ChIP-seq were used for enrichment analysis using curated MSigDB gene sets (FDR cutoff>0.05).

Supplementary Table S1. **Proliferation score and Risk of Recurrence (ROR) scores.**

Supplementary Table S2. **Resultant gene list for all enrichment analysis**

Supplementary Table S3. **Description of selected upregulated genes in low *DAB2IP* Luminal A breast cancer tumors.** Fold-changes are DAB2IP high relative to DAB2IP low, hence log2FC are negative.

Supplementary Table S4. **Breast cancer patient cohort information with different clinicopathological factors.**

Supplementary Table S5. **Up- and down-regulated NF-κB genes in *DAB2IP*-low Luminal A tumors.**

Supplementary Table S6. **Neojunction counts in Luminal A and Luminal B tumors by *DAB2IP* high/low levels.**

Supplementary Table S7. **Description of all NF-κB motifs analyzed for the TCGA *DAB2IP*-low Luminal A breast cancer cohort and up and down peak associated genes between shDAB2IP and shControl T47D cells for RELA, RELB, and NFKB2.**

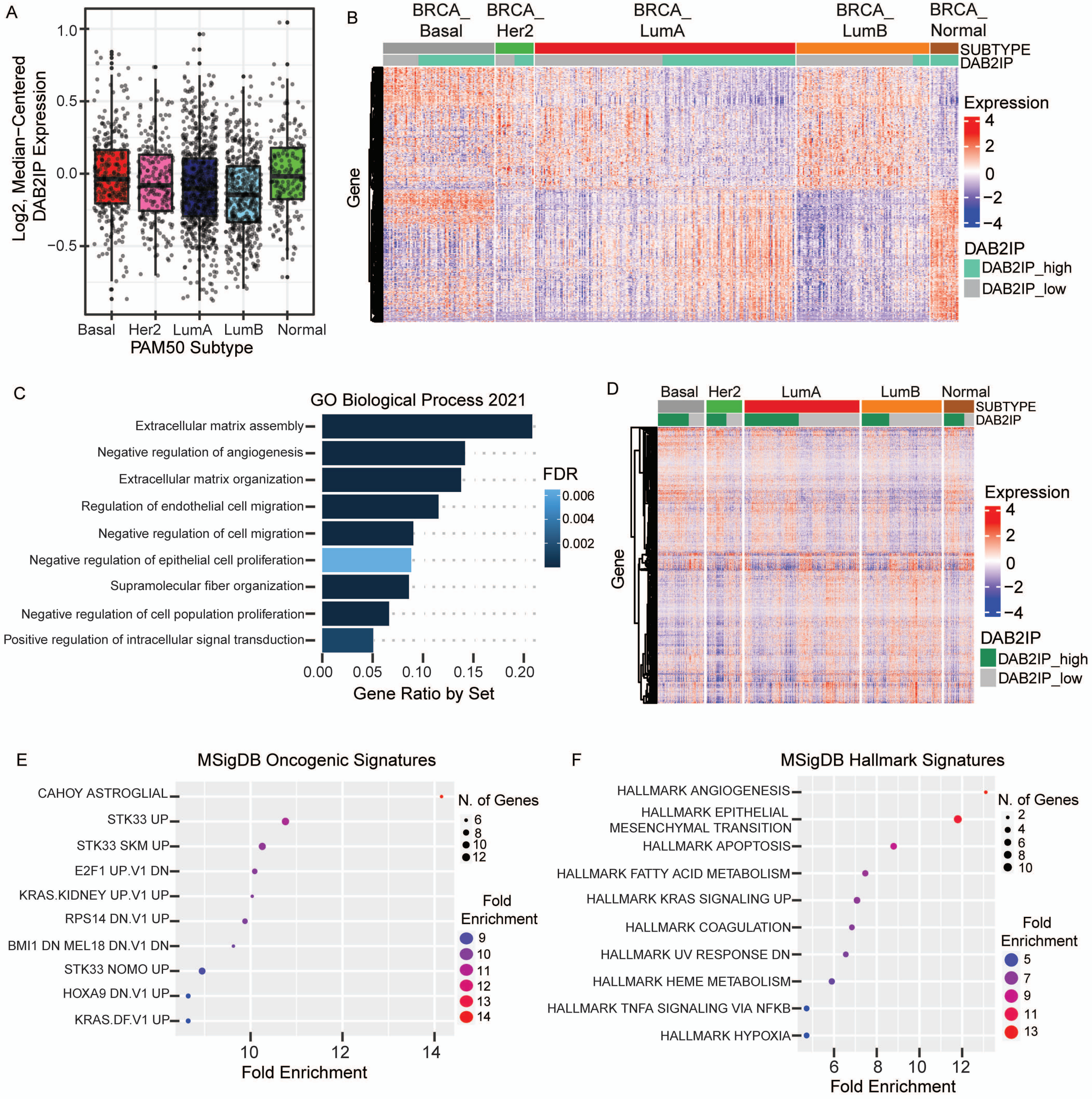
A
Log2, Median−Centered DAB2IP Expression
PAM50 Subtype
Basal
Her2
LumA
LumB
Normal
B
BRCA_ Basal
BRCA_ Her2
BRCA_ LumA
BRCA_ LumB
BRCA_ Normal
SUBTYPE
DAB2IP
Gene
Expression
DAB2IP_high
DAB2IP_low
C
GO Biological Process 2021
Extracellular matrix assembly
Negative regulation of angiogenesis
Extracellular matrix organization
Regulation of endothelial cell migration
Negative regulation of cell migration
Negative regulation of epithelial cell proliferation
Supramolecular fiber organization
Negative regulation of cell population proliferation
Positive regulation of intracellular signal transduction
FDR
Gene Ratio by Set
D
Basal
Her2
LumA
LumB
Normal
SUBTYPE
DAB2IP
Gene
Expression
DAB2IP_high
DAB2IP_low
E
MSigDB Oncogenic Signatures
CAHOY ASTROGLIAL
STK33 UP
STK33 SKM UP
E2F1 UP.V1 DN
KRAS.KIDNEY UP.V1 UP
RPS14 DN.V1 UP
BMI1 DN MEL18 DN.V1 DN
STK33 NOMO UP
HOXA9 DN.V1 UP
KRAS.DF.V1 UP
N. of Genes
Fold Enrichment
F
MSigDB Hallmark Signatures
HALLMARK ANGIOGENESIS
HALLMARK EPITHELIAL MESENCHYMAL TRANSITION
HALLMARK APOPTOSIS
HALLMARK FATTY ACID METABOLISM
HALLMARK KRAS SIGNALING UP
HALLMARK COAGULATION
HALLMARK UV RESPONSE DN
HALLMARK HEME METABOLISM
HALLMARK TNFA SIGNALING VIA NFKB
HALLMARK HYPOXIA
N. of Genes
Fold Enrichment

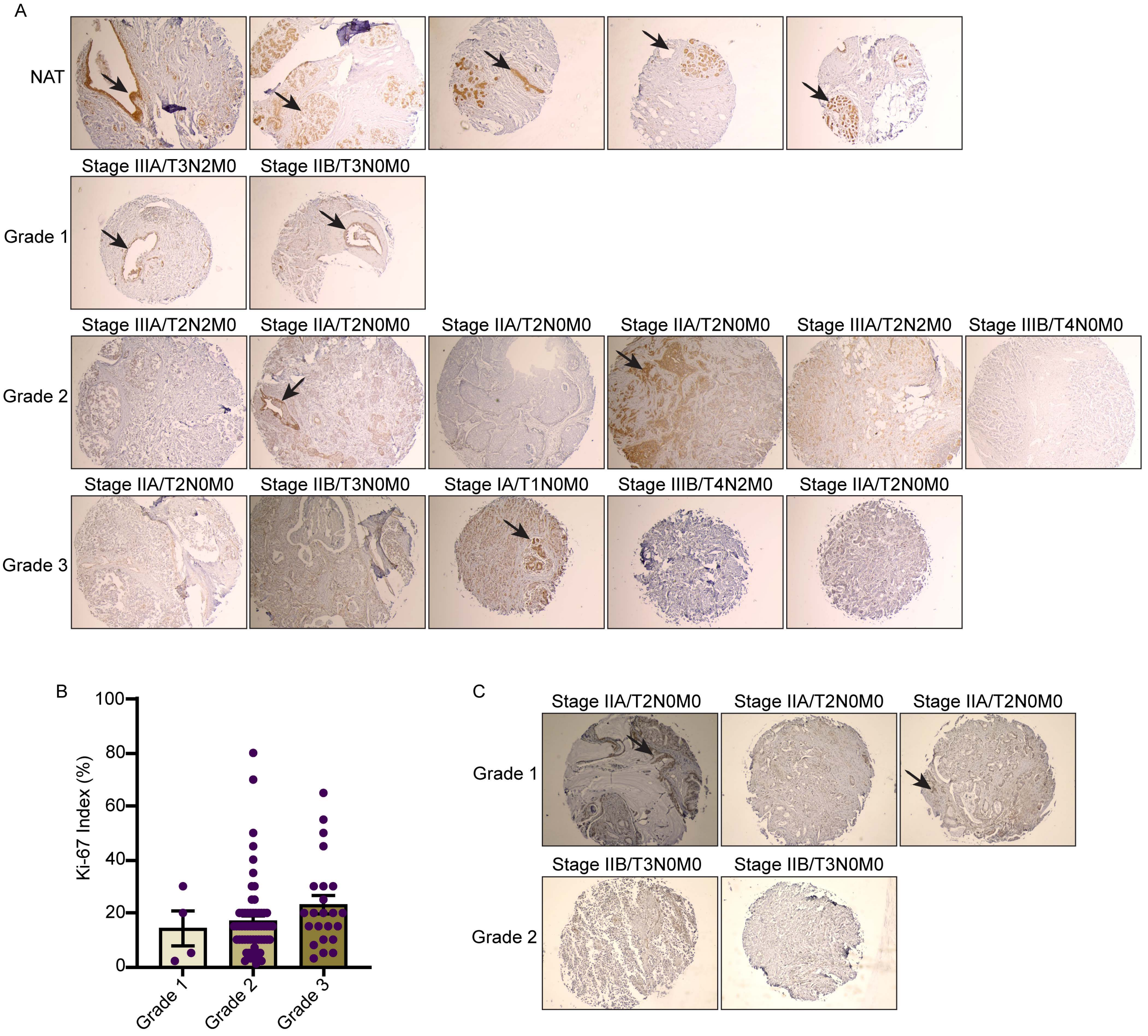
A
NAT
Stage IIIA/T3N2M0
Stage IIB/T3N0M0
Grade 1
Stage IIIA/T2N2M0
Stage IIA/T2N0M0
Stage IIA/T2N0M0
Stage IIA/T2N0M0
Stage IIIA/T2N2M0
Stage IIIB/T4N0M0
Grade 2
Stage IIA/T2N0M0
Stage IIB/T3N0M0
Stage IA/T1N0M0
Stage IIIB/T4N2M0
Stage IIA/T2N0M0
Grade 3
B
Ki-67 Index (%)
100
80
60
40
20
0
Grade 1
Grade 2
Grade 3
C
Stage IIA/T2N0M0
Stage IIA/T2N0M0
Stage IIA/T2N0M0
Grade 1
Stage IIB/T3N0M0
Stage IIB/T3N0M0
Grade 2

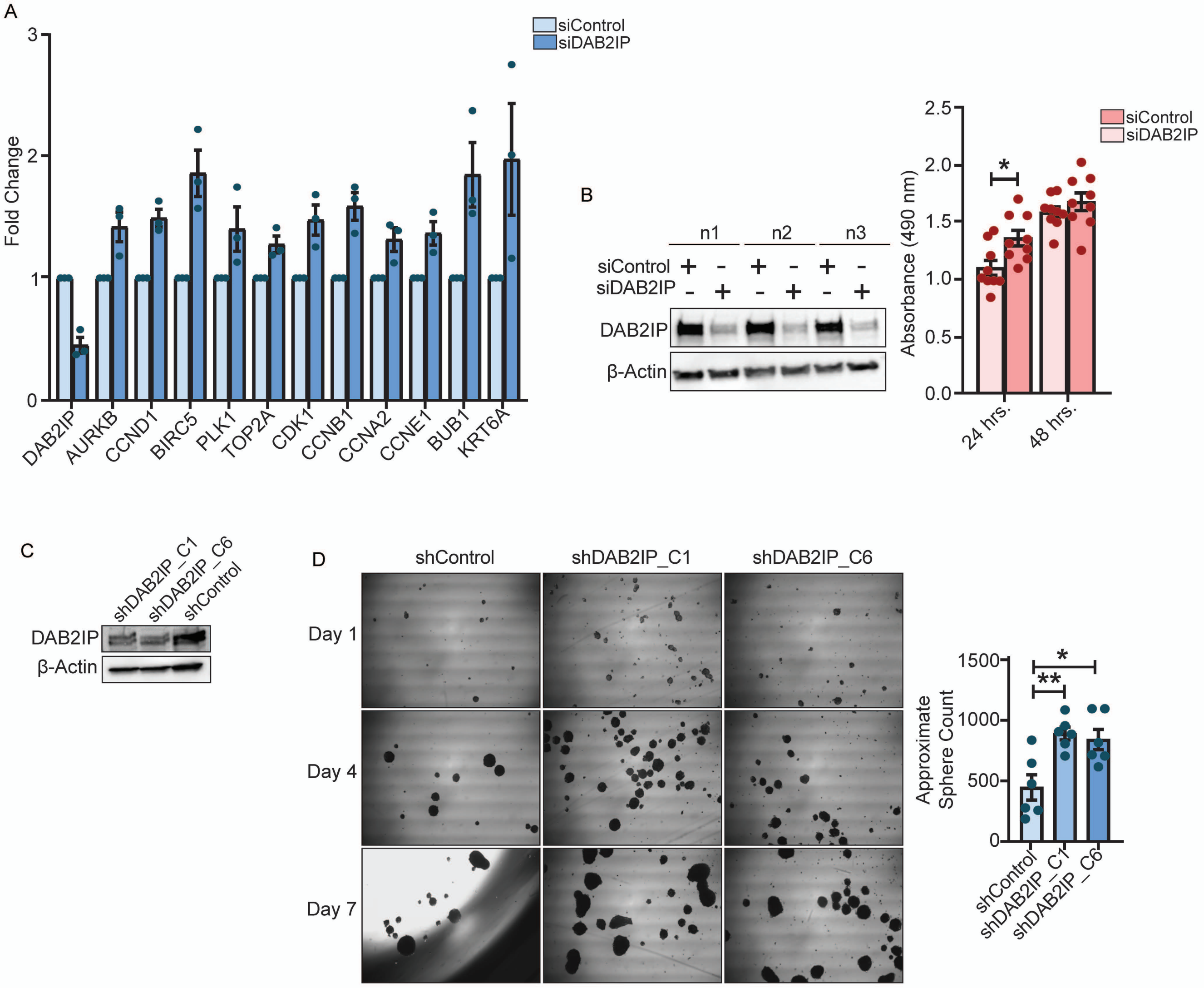
A
siControl
siDAB2IP
Fold Change
3
2
1
0
DAB2IP
AURKB
CCND1
BIRC5
PLK1
TOP2A
CDK1
CCNB1
CCNA2
CCNE1
BUB1
KRT6A
B
n1
n2
n3
siControl + - + - + -
siDAB2IP - + - + - +
DAB2IP
β-Actin
siControl
siDAB2IP
Absorbance (490 nm)
2.5
2.0
1.5
1.0
0.5
0.0
*
24 hrs.
48 hrs.
C
shDAB2IP_C1
shDAB2IP_C6
shControl
DAB2IP
β-Actin
D
shControl
shDAB2IP_C1
shDAB2IP_C6
Day 1
Day 4
Day 7
Approximate Sphere Count
1500
1000
500
0
*
**
shControl
shDAB2IP_C1
shDAB2IP_C6

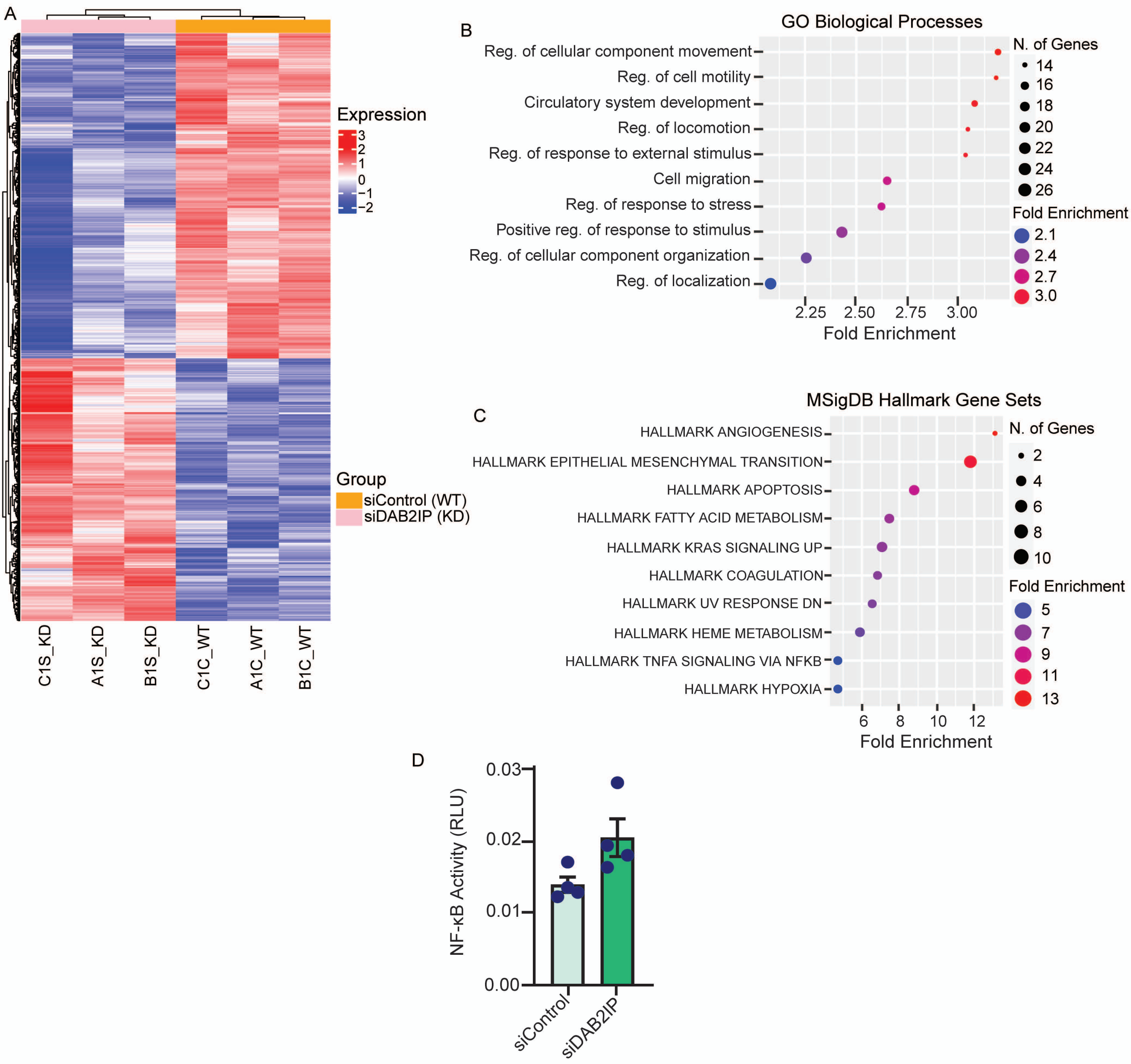

A
Expression
3
2
1
0
−1
−2
Group
siControl (WT)
siDAB2IP (KD)
C1S_KD
A1S_KD
B1S_KD
C1C_WT
A1C_WT
B1C_WT
B
GO Biological Processes
Reg. of cellular component movement
Reg. of cell motility
Circulatory system development
Reg. of locomotion
Reg. of response to external stimulus
Cell migration
Reg. of response to stress
Positive reg. of response to stimulus
Reg. of cellular component organization
Reg. of localization
2.25
2.50
2.75
3.00
Fold Enrichment
N. of Genes
14
16
18
20
22
24
26
Fold Enrichment
2.1
2.4
2.7
3.0
C
MSigDB Hallmark Gene Sets
HALLMARK ANGIOGENESIS
HALLMARK EPITHELIAL MESENCHYMAL TRANSITION
HALLMARK APOPTOSIS
HALLMARK FATTY ACID METABOLISM
HALLMARK KRAS SIGNALING UP
HALLMARK COAGULATION
HALLMARK UV RESPONSE DN
HALLMARK HEME METABOLISM
HALLMARK TNFA SIGNALING VIA NFKB
HALLMARK HYPOXIA
6
8
10
12
Fold Enrichment
N. of Genes
2
4
6
8
10
Fold Enrichment
5
7
9
11
13
D
0.03
0.02
0.01
0.00
NF-κB Activity (RLU)
siControl
siDAB2IP

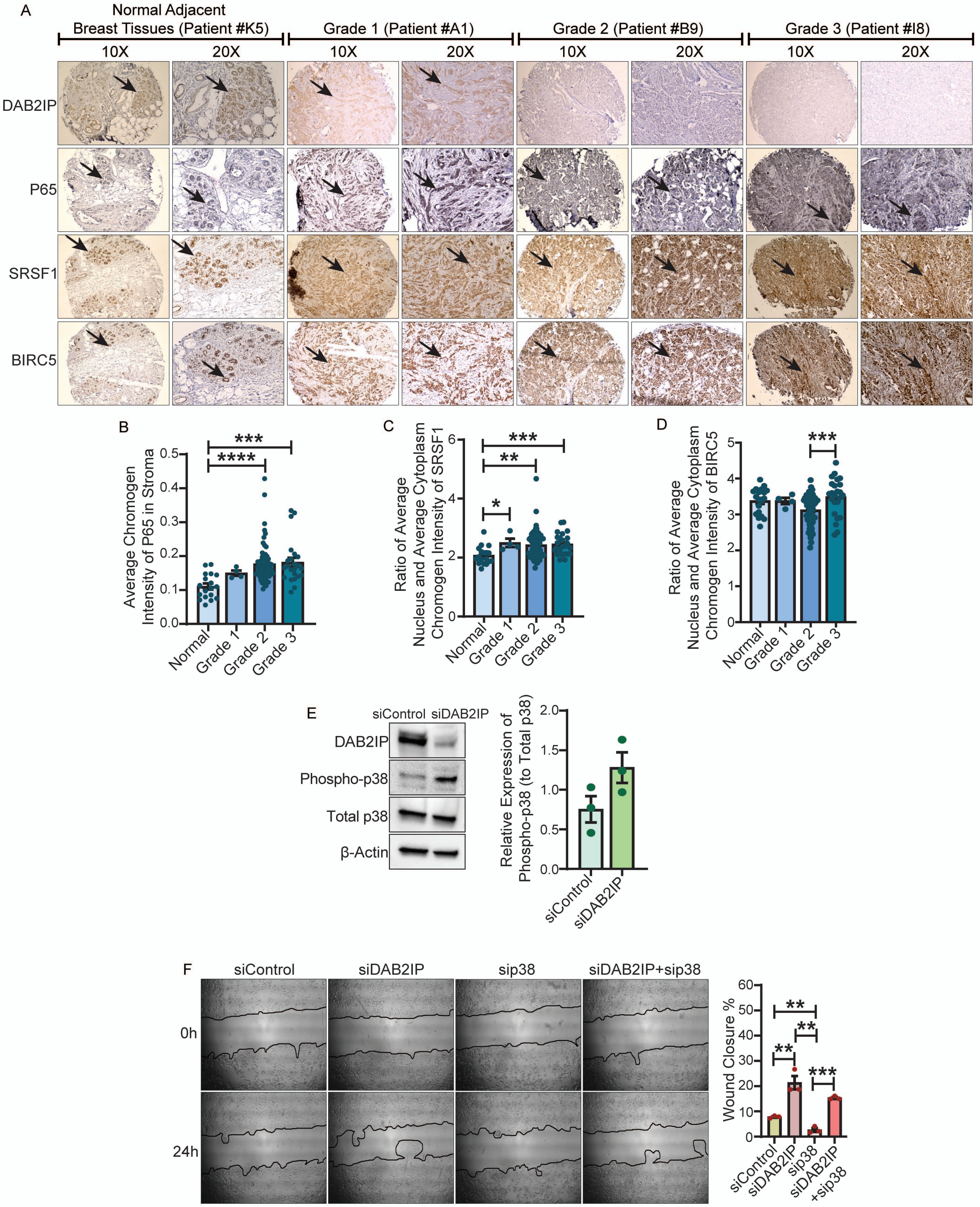

A
Normal Adjacent Breast Tissues (Patient #K5)
Grade 1 (Patient #A1)
Grade 2 (Patient #B9)
Grade 3 (Patient #I8)
10X
20X
DAB2IP
P65
SRSF1
BIRC5
B
Average Chromogen Intensity of P65 in Stroma
Normal
Grade 1
Grade 2
Grade 3
C
Ratio of Average Nucleus and Average Cytoplasm Chromogen Intensity of SRSF1
D
Ratio of Average Nucleus and Average Cytoplasm Chromogen Intensity of BIRC5
E
siControl
siDAB2IP
DAB2IP
Phospho-p38
Total p38
β-Actin
Relative Expression of Phospho-p38 (to Total p38)
F
siControl
siDAB2IP
sip38
siDAB2IP+sip38
0h
24h
Wound Closure %

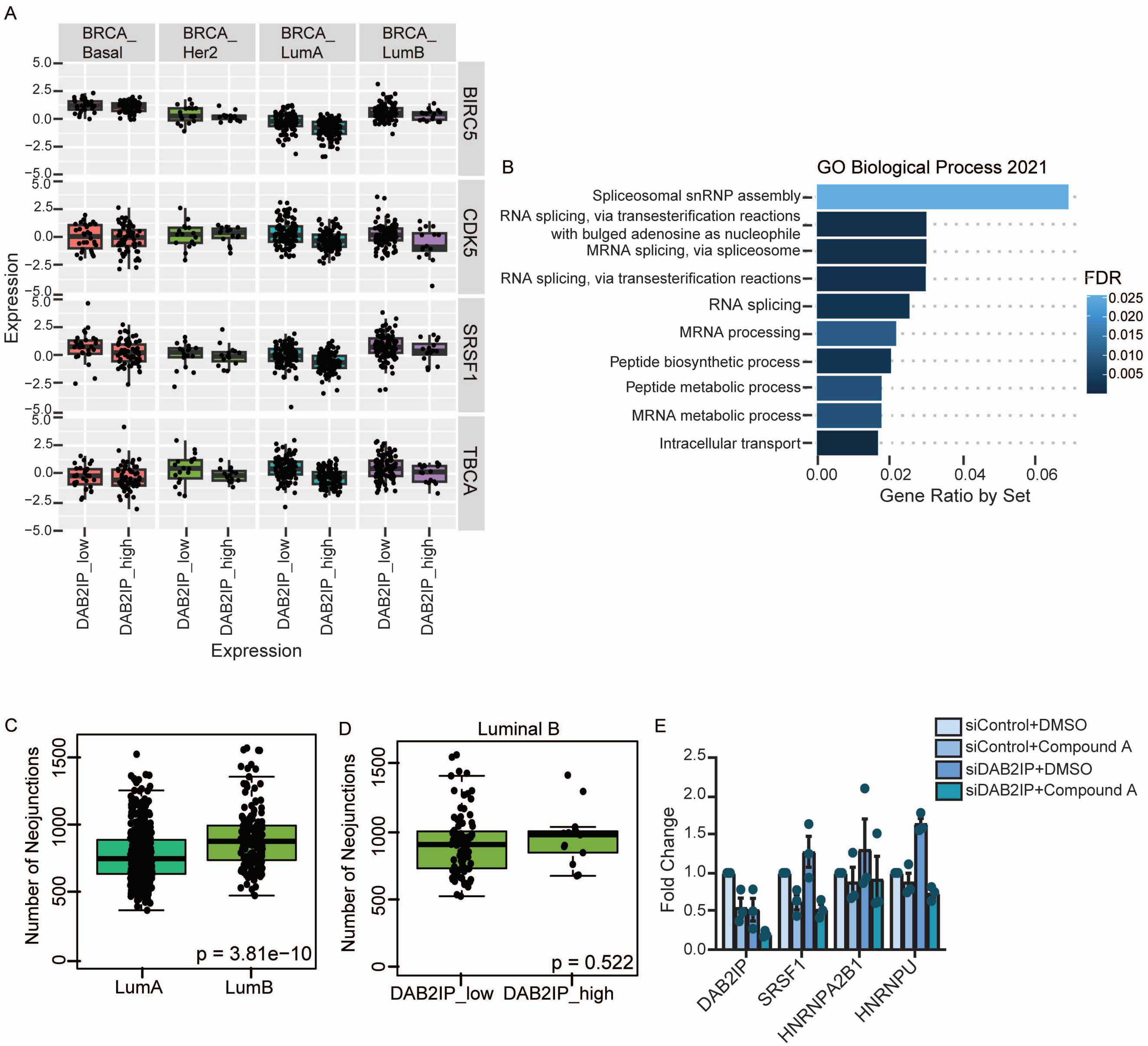
A
BRCA_ Basal
BRCA_ Her2
BRCA_ LumA
BRCA_ LumB
BIRC5
CDK5
SRSF1
TBCA
Expression
DAB2IP_low
DAB2IP_high
B
GO Biological Process 2021
Spliceosomal snRNP assembly
RNA splicing, via transesterification reactions with bulged adenosine as nucleophile
MRNA splicing, via spliceosome
RNA splicing, via transesterification reactions
RNA splicing
MRNA processing
Peptide biosynthetic process
Peptide metabolic process
MRNA metabolic process
Intracellular transport
Gene Ratio by Set
FDR
C
Number of Neojunctions
LumA
LumB
p = 3.81e−10
D
Luminal B
p = 0.522
E
siControl+DMSO
siControl+Compound A
siDAB2IP+DMSO
siDAB2IP+Compound A
Fold Change
DAB2IP
SRSF1
HNRNPA2B1
HNRNPU

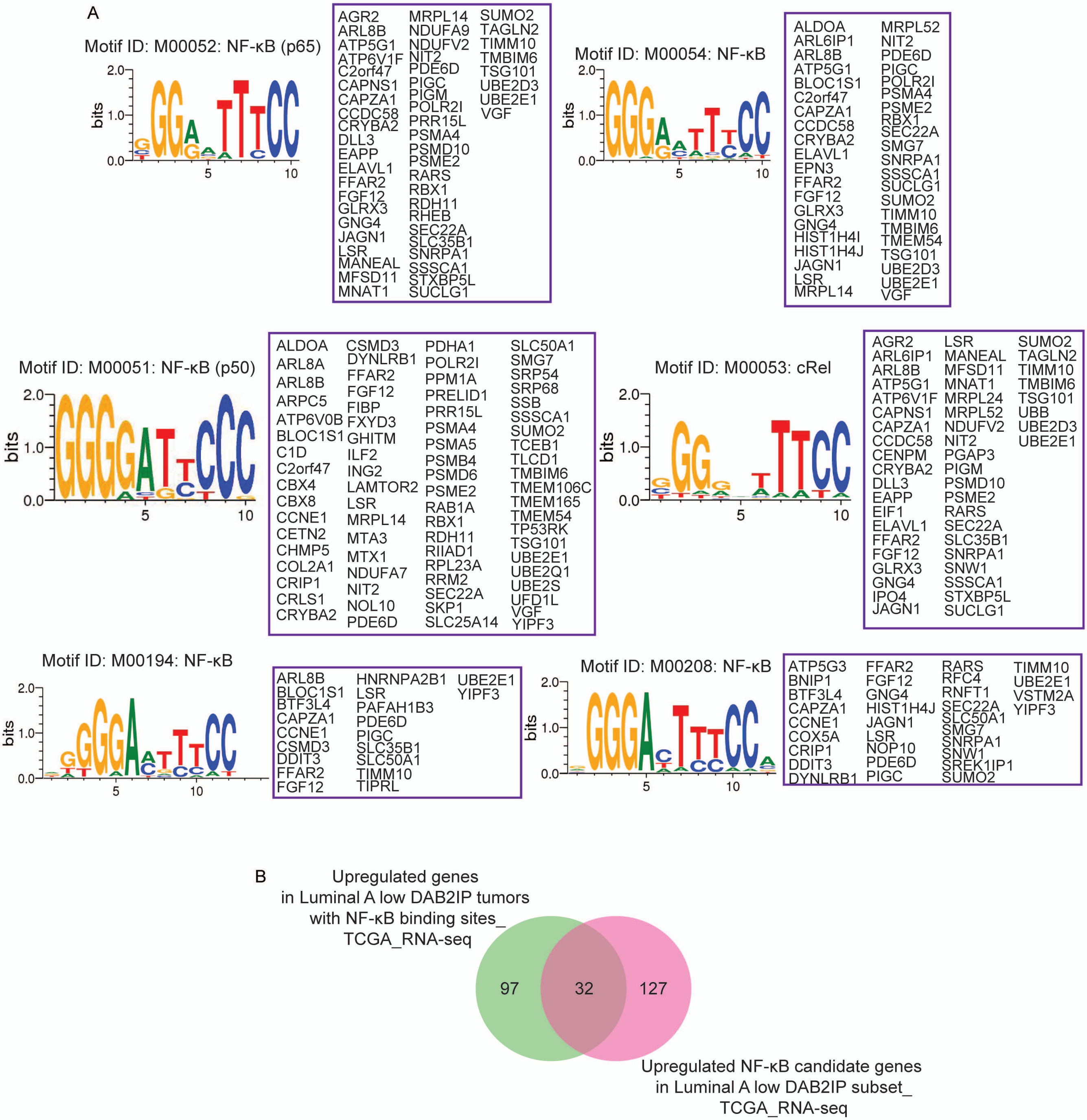

A
Motif ID: M00052: NF-κB (p65)
bits
AGR2
ARL8B
ATP5G1
ATP6V1F
C2orf47
CAPNS1
CAPZA1
CCDC58
CRYBA2
DLL3
EAPP
ELAVL1
FFAR2
FGF12
GLRX3
GNG4
JAGN1
LSR
MANEAL
MFSD11
MNAT1
MRPL14
NDUFA9
NDUFV2
NIT2
PDE6D
PIGC
PIGM
POLR2I
PRR15L
PSMA4
PSMD10
PSME2
RARS
RBX1
RDH11
RHEB
SEC22A
SLC35B1
SNRPA1
SSSCA1
STXBP5L
SUCLG1
SUMO2
TAGLN2
TIMM10
TMBIM6
TSG101
UBE2D3
UBE2E1
VGF
Motif ID: M00054: NF-κB
bits
ALDOA
ARL6IP1
ARL8B
ATP5G1
BLOC1S1
C2orf47
CAPZA1
CCDC58
CRYBA2
ELAVL1
EPN3
FFAR2
FGF12
GLRX3
GNG4
HIST1H4I
HIST1H4J
JAGN1
LSR
MRPL14
MRPL52
NIT2
PDE6D
PIGC
POLR2I
PSMA4
PSME2
RBX1
SEC22A
SMG7
SNRPA1
SSSCA1
SUCLG1
SUMO2
TIMM10
TMBIM6
TMEM54
TSG101
UBE2D3
UBE2E1
VGF
Motif ID: M00051: NF-κB (p50)
bits
ALDOA
ARL8A
ARL8B
ARPC5
ATP6V0B
BLOC1S1
C1D
C2orf47
CBX4
CBX8
CCNE1
CETN2
CHMP5
COL2A1
CRIP1
CRLS1
CRYBA2
CSMD3
DYNLRB1
FFAR2
FGF12
FIBP
FXYD3
GHITM
ILF2
ING2
LAMTOR2
LSR
MRPL14
MTA3
MTX1
NDUFA7
NIT2
NOL10
PDE6D
PDHA1
POLR2I
PPM1A
PRELID1
PRR15L
PSMA4
PSMA5
PSMB4
PSMD6
PSME2
RAB1A
RBX1
RDH11
RIIAD1
RPL23A
RRM2
SEC22A
SKP1
SLC25A14
SLC50A1
SMG7
SRP54
SRP68
SSB
SSSCA1
SUMO2
TCEB1
TLCD1
TMBIM6
TMEM106C
TMEM165
TMEM54
TP53RK
TSG101
UBE2E1
UBE2Q1
UBE2S
UFD1L
VGF
YIPF3
Motif ID: M00053: cRel
bits
AGR2
ARL6IP1
ARL8B
ATP5G1
ATP6V1F
CAPNS1
CAPZA1
CCDC58
CENPM
CRYBA2
DLL3
EAPP
EIF1
ELAVL1
FFAR2
FGF12
GLRX3
GNG4
IPO4
JAGN1
LSR
MANEAL
MFSD11
MNAT1
MRPL24
MRPL52
NDUFV2
NIT2
PGAP3
PIGM
PSMD10
PSME2
RARS
SEC22A
SLC35B1
SNRPA1
SNW1
SSSCA1
STXBP5L
SUCLG1
SUMO2
TAGLN2
TIMM10
TMBIM6
TSG101
UBB
UBE2D3
UBE2E1
Motif ID: M00194: NF-κB
bits
ARL8B
BLOC1S1
BTF3L4
CAPZA1
CCNE1
CSMD3
DDIT3
FFAR2
FGF12
HNRNPA2B1
LSR
PAFAH1B3
PDE6D
PIGC
SLC35B1
SLC50A1
TIMM10
TIPRL
UBE2E1
YIPF3
Motif ID: M00208: NF-κB
bits
ATP5G3
BNIP1
BTF3L4
CAPZA1
CCNE1
COX5A
CRIP1
DDIT3
DYNLRB1
FFAR2
FGF12
GNG4
HIST1H4J
JAGN1
LSR
NOP10
PDE6D
PIGC
RARS
RFC4
RNFT1
SEC22A
SLC50A1
SMG7
SNRPA1
SNW1
SREK1IP1
SUMO2
TIMM10
UBE2E1
VSTM2A
YIPF3
B
Upregulated genes in Luminal A low DAB2IP tumors with NF-κB binding sites_ TCGA_RNA-seq
97
32
127
Upregulated NF-κB candidate genes in Luminal A low DAB2IP subset_ TCGA_RNA-seq

A

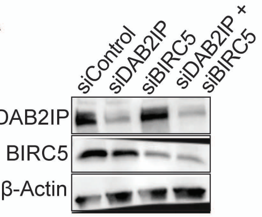
siControl
siDAB2IP
siBIRC5
siDAB2IP +
siBIRC5
DAB2IP
BIRC5
β-Actin

B

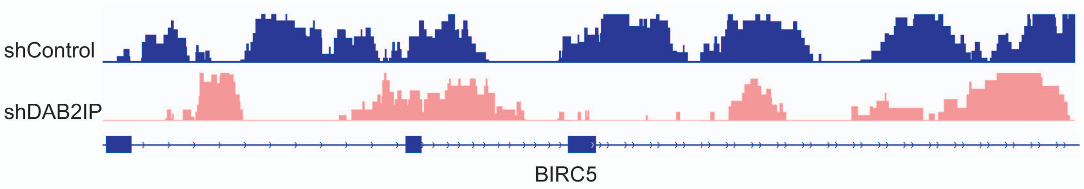
shControl
shDAB2IP
BIRC5

C

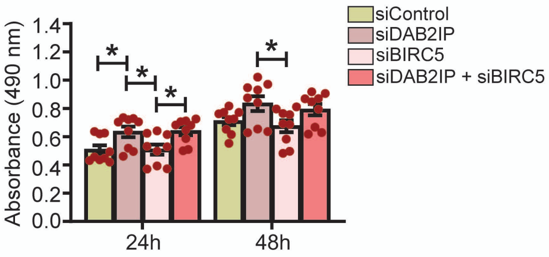
Absorbance (490 nm)
1.4
1.2
1.0
0.8
0.6
0.4
0.2
0.0
24h
48h
*
siControl
siDAB2IP
siBIRC5
siDAB2IP + siBIRC5

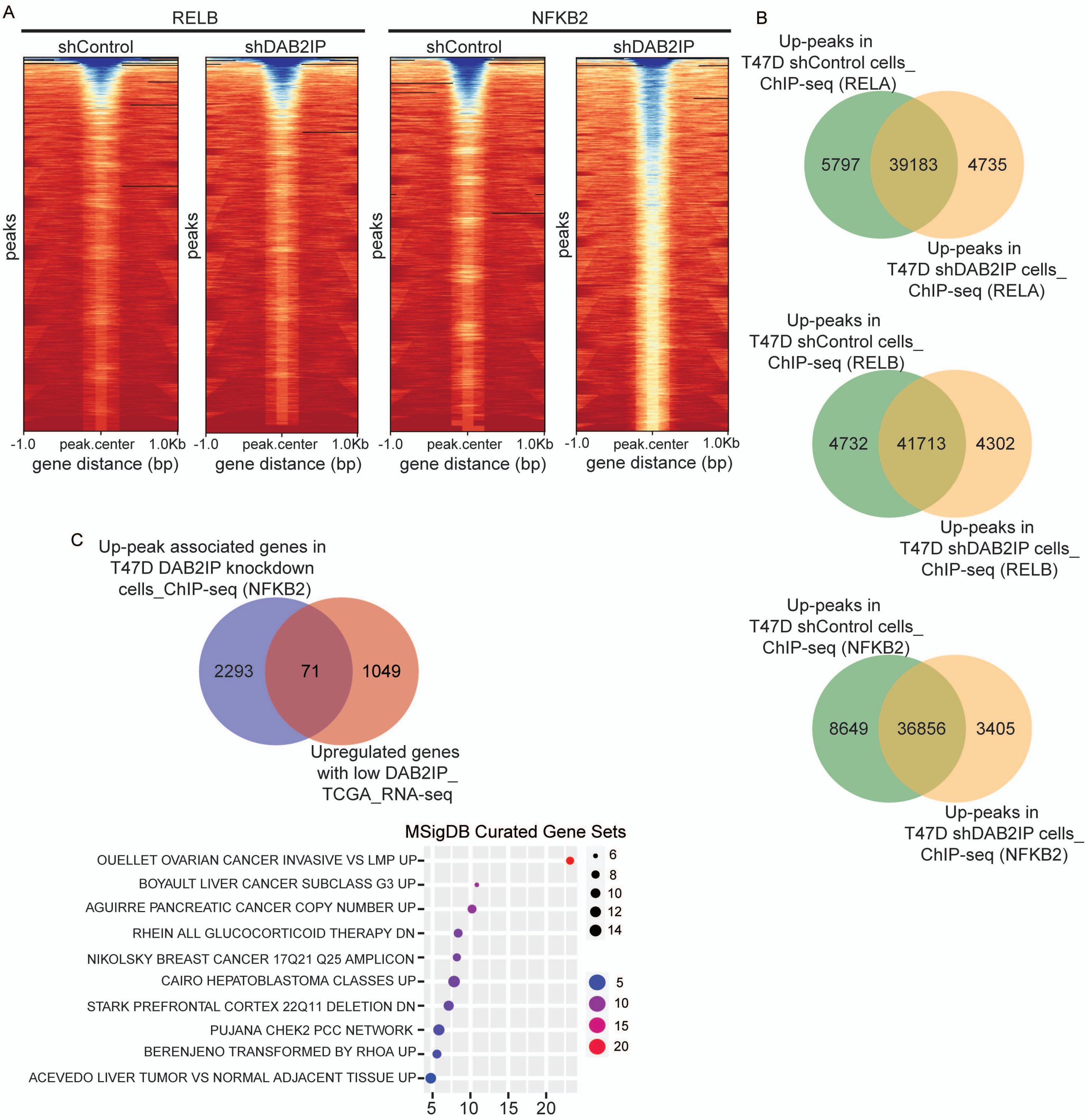
A
RELB
NFKB2
shControl
shDAB2IP
shControl
shDAB2IP
peaks
-1.0 peak.center 1.0Kb
gene distance (bp)
B
Up-peaks in T47D shControl cells_ ChIP-seq (RELA)
5797
39183
4735
Up-peaks in T47D shDAB2IP cells_ ChIP-seq (RELA)
Up-peaks in T47D shControl cells_ ChIP-seq (RELB)
4732
41713
4302
Up-peaks in T47D shDAB2IP cells_ ChIP-seq (RELB)
Up-peaks in T47D shControl cells_ ChIP-seq (NFKB2)
8649
36856
3405
Up-peaks in T47D shDAB2IP cells_ ChIP-seq (NFKB2)
C
Up-peak associated genes in T47D DAB2IP knockdown cells_ChIP-seq (NFKB2)
2293
71
1049
Upregulated genes with low DAB2IP_ TCGA_RNA-seq
MSigDB Curated Gene Sets
OUELLET OVARIAN CANCER INVASIVE VS LMP UP
BOYAULT LIVER CANCER SUBCLASS G3 UP
AGUIRRE PANCREATIC CANCER COPY NUMBER UP
RHEIN ALL GLUCOCORTICOID THERAPY DN
NIKOLSKY BREAST CANCER 17Q21 Q25 AMPLICON
CAIRO HEPATOBLASTOMA CLASSES UP
STARK PREFRONTAL CORTEX 22Q11 DELETION DN
PUJANA CHEK2 PCC NETWORK
BERENJENO TRANSFORMED BY RHOA UP
ACEVEDO LIVER TUMOR VS NORMAL ADJACENT TISSUE UP
5 10 15 20
6
8
10
12
14
5
10
15
20
