# Supplementary material for: DAB2IP loss in luminal a breast cancer leads to NF-κB–associated aggressive oncogenic phenotypes
Source: JCI Insight. 2024 Dec 6;9(23):e171705. doi: 10.1172/jci.insight.171705 (PMC11623953; doi:10.1172/jci.insight.171705)

Full unedited gel for Figure 4B T47D – Knockdown efficiency of DAB2IP via siRNA

siControl
siDAB2IP

Anti-DAB2IP

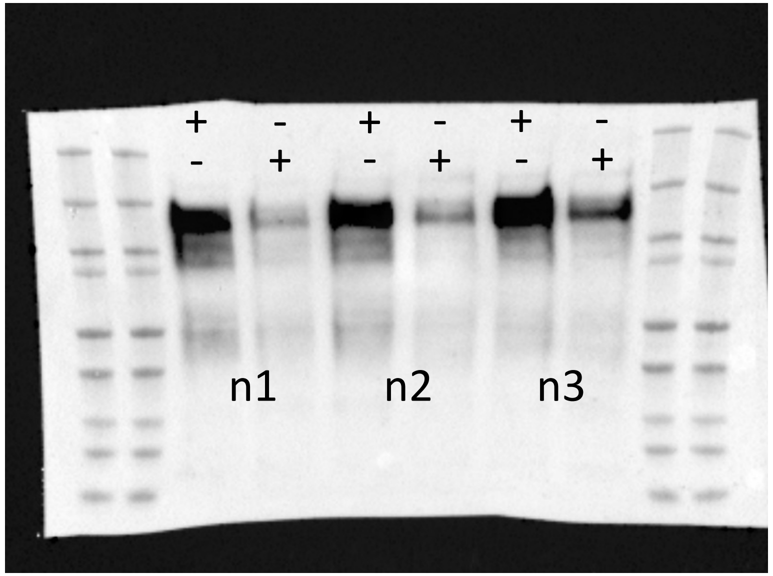

Anti-β-actin

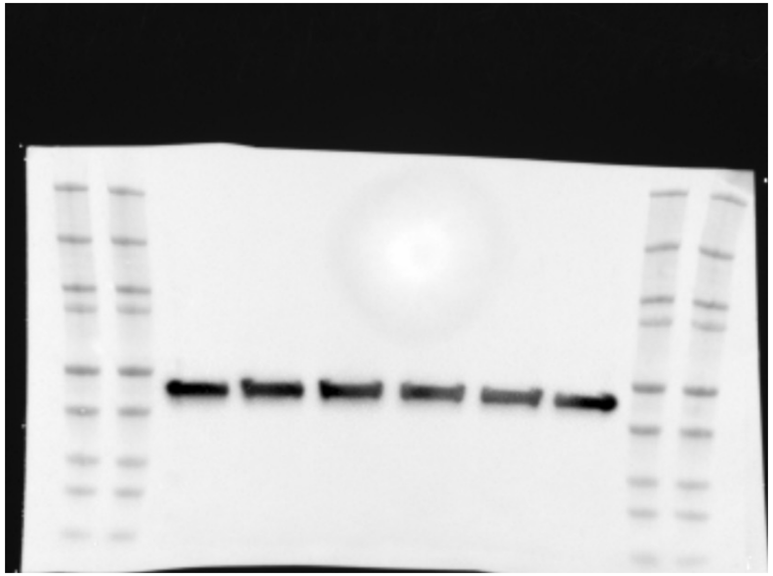

Full unedited gel for Figure 4D

T47D – Knockdown efficiency of DAB2IP via shRNA

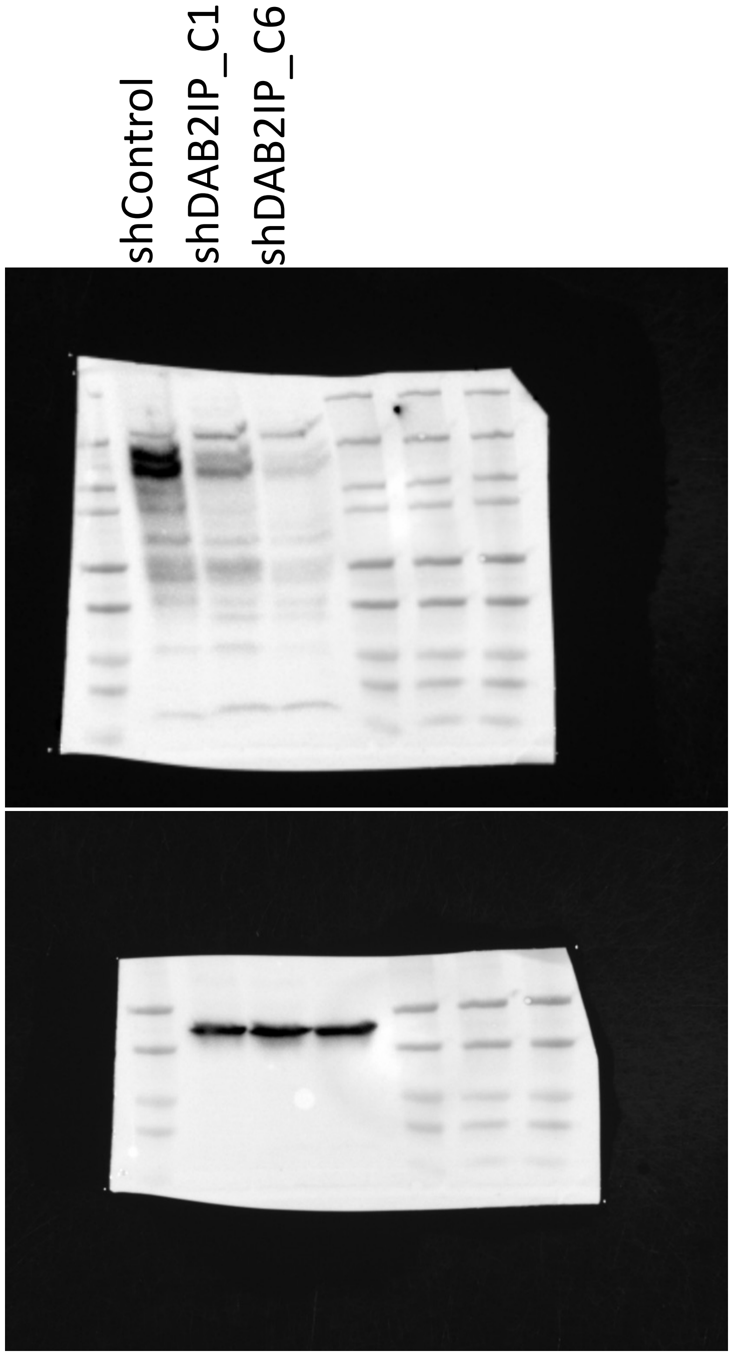

Full unedited gel for Figure 5B

MCF10A – Knockdown efficiency of DAB2IP via siRNA
&
Expression of Phosho-IKKαβ in MCF10A siDAB2IP and siControl cells

Lane 1: siControl] n1
Lane 2: siDAB2IP]
Lane 3: siControl] n2
Lane 4: siDAB2IP]
Lane 5: siControl] n3
Lane 6: siDAB2IP]
Lane 7: siControl] n4
Lane 8: siDAB2IP]

Lane 1 & 2: n1 – very faint band in lane 1 (siControl). Performed experiments with n2, & n3 and n4 lysates. Figure 5B is blot from n3

Anti-DAB2IP

Anti-β-actin

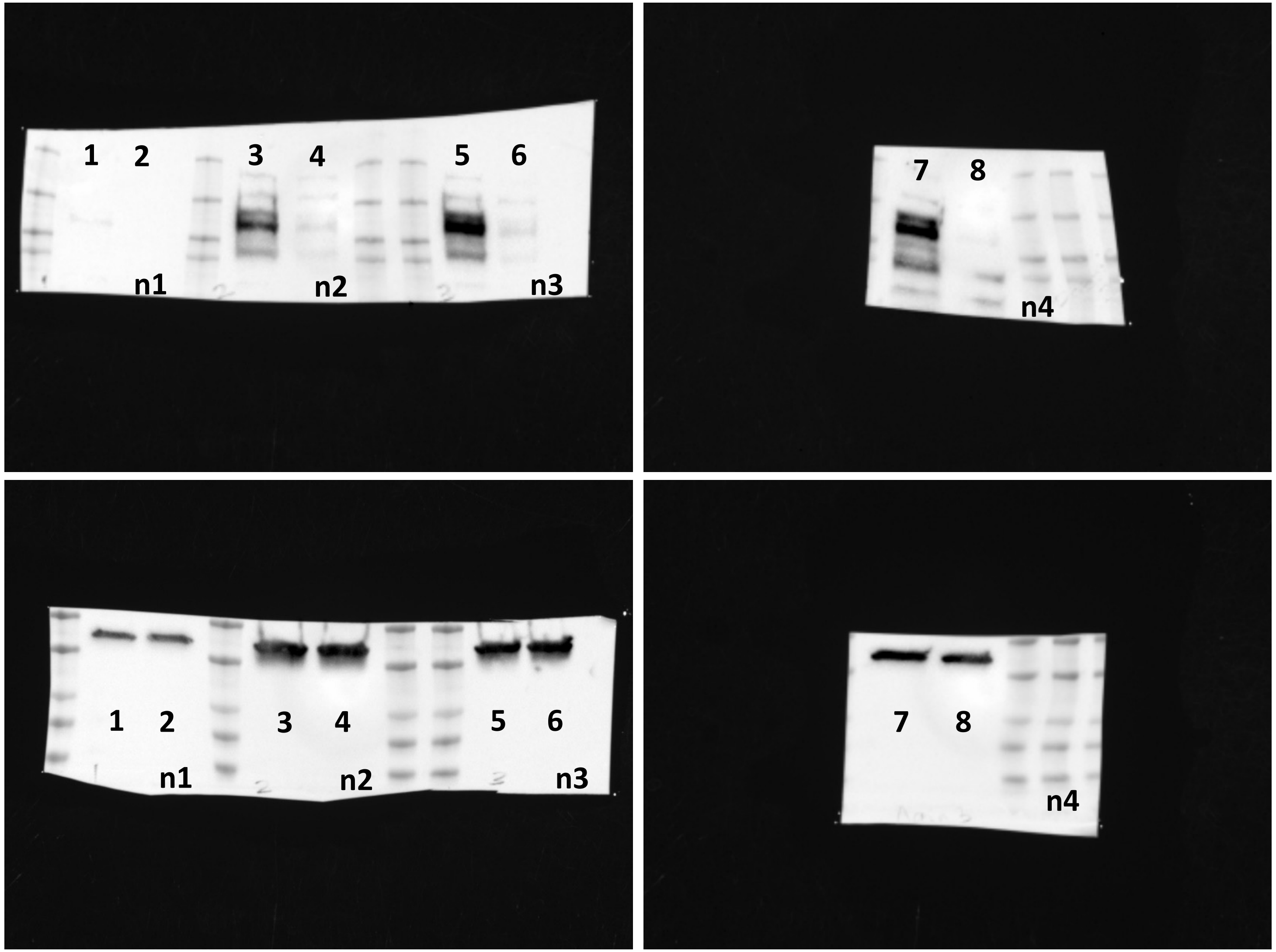

Full unedited gel for Figure 5B

MCF10A – Knockdown efficiency of DAB2IP via siRNA &
Expression of Phosho-IKKαβ in MCF10A siDAB2IP and siControl cells

Anti-Phospho-IKKαβ

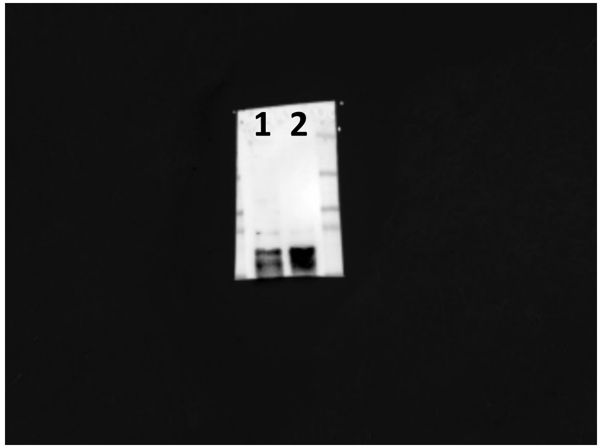

Anti-IKKα

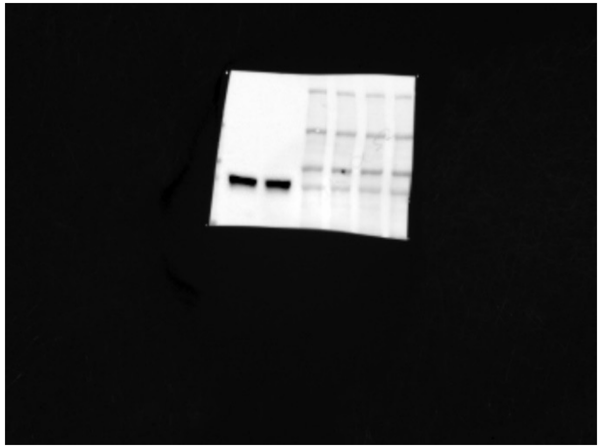

Anti-IKKβ

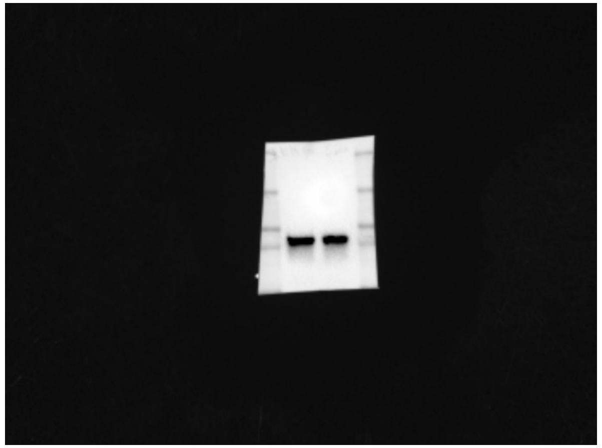

Anti-β-actin

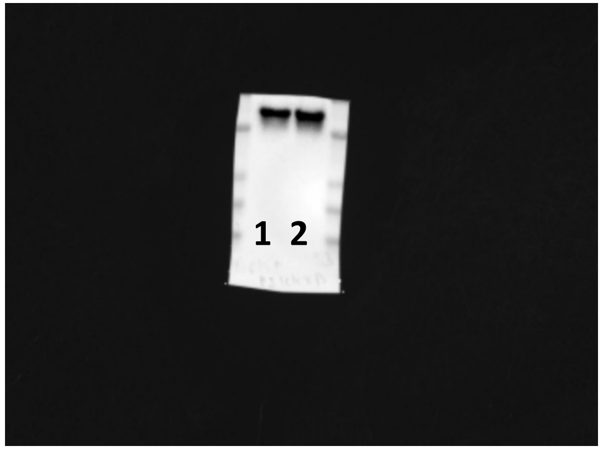

Anti-β-actin

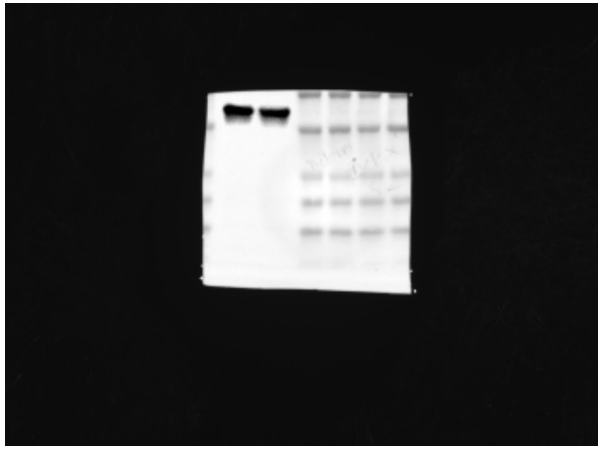

Anti-β-actin

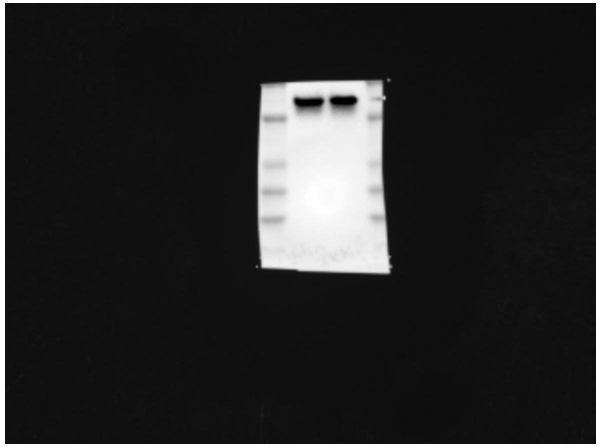

Lane 1: siControl
Lane 2: siDAB2IP

Used same lysates in the same gel but 3 times in a 15-well gel and cut the blots for 3 separate primary antibodies and corresponding loading control

Full unedited gel for Figure 5E

T47D - Expression of Phosho-IKKαβ in T47D siDAB2IP and siControl cells (nuclear fractionation)

Anti-DAB2IP

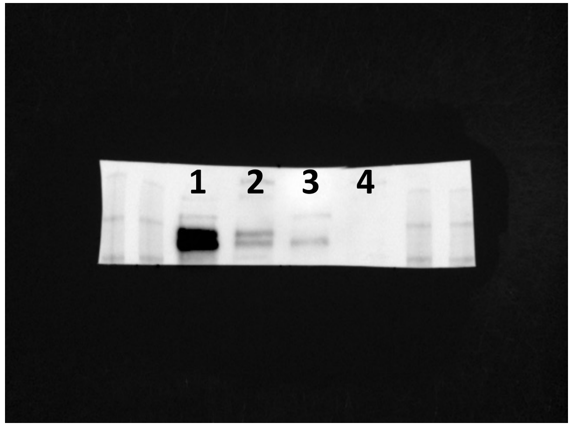

Anti-Phospho-IKKαβ

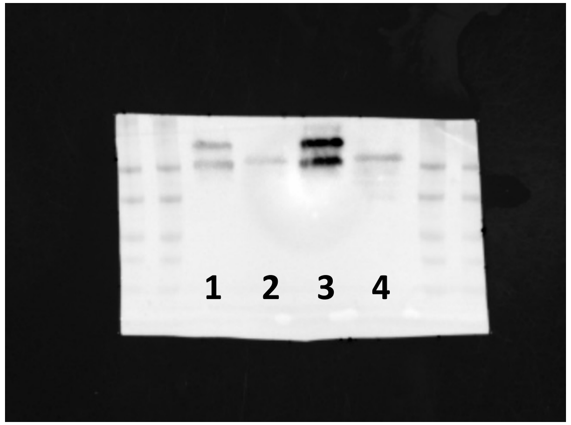

Anti-Phospho-IKKαβ (Longer Exposure)

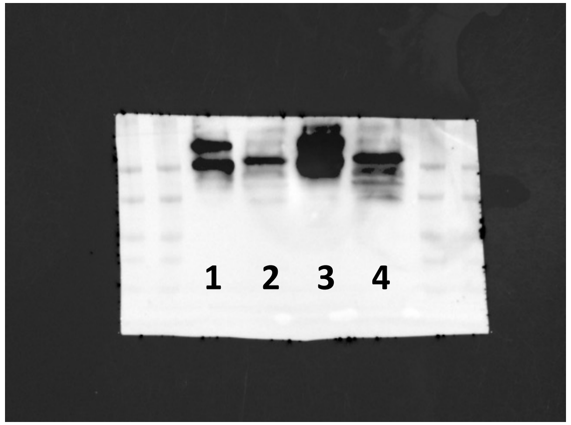

β-tubulin

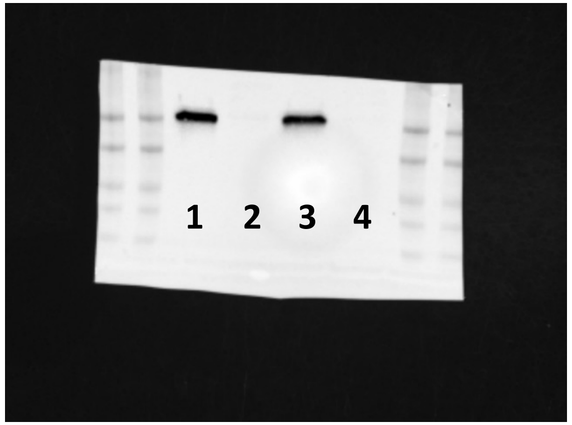

Lamin A/C

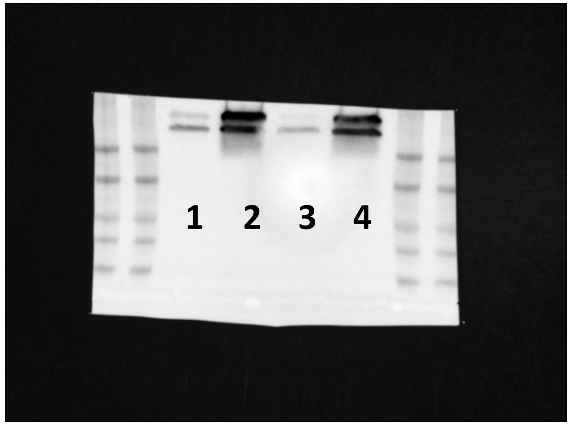

Lane 1: siControl – Cytoplasmic extract
Lane 2: siControl – Nuclear extract
Lane 3: siDAB2IP – Cytoplasmic extract
Lane 4: siDAB2IP – Nuclear extract

Full unedited gel for Figure 6D

T47D – Knockdown efficiency of DAB2IP, SRSF1 via siRNA. Increased expression of SRSF1 with knockdown of DAB2IP

Blot was cut for incubation in antibodies

Lane 1: siControl

Lane 2: siDAB2IP

Lane 3: siSRSF1

Lane 4: siDAB2IP + siSRSF1

Anti-DAB2IP

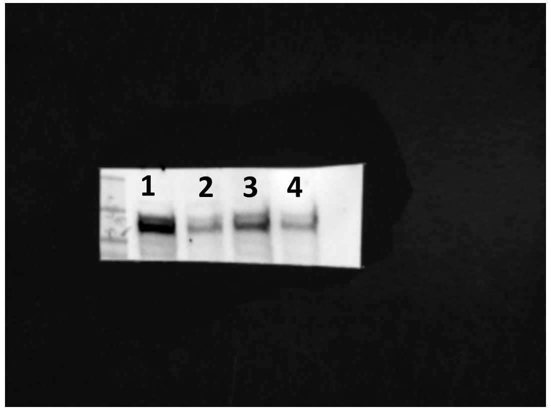

Anti-SRSF1

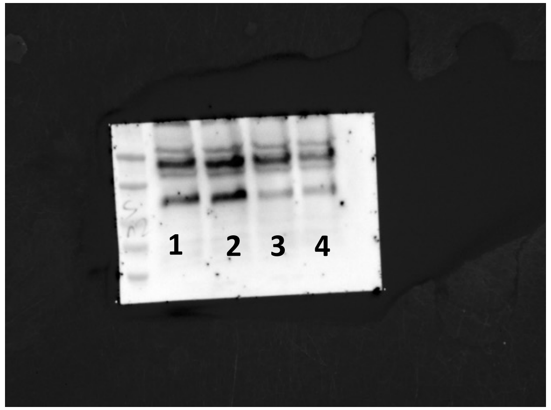

Anti-β-actin

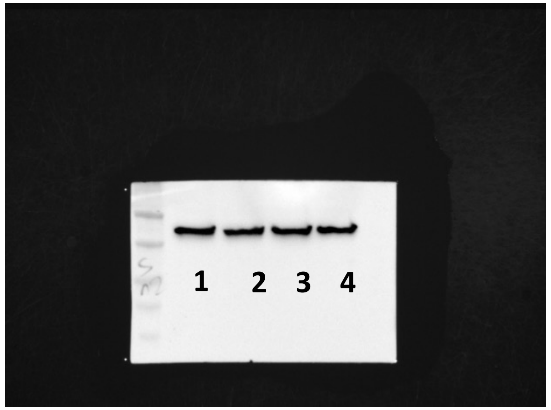

Mol. wt of SRSF1 is 33 kDA, hence consider the lower band which also reduces substantially with knockdown

Full unedited gel for Suppl. Figure S3B

BT474 – Knockdown efficiency of DAB2IP via siRNA

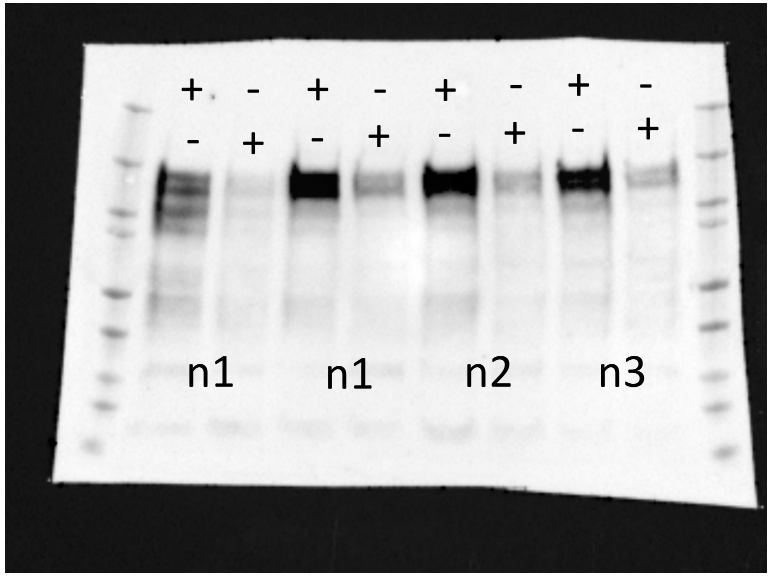

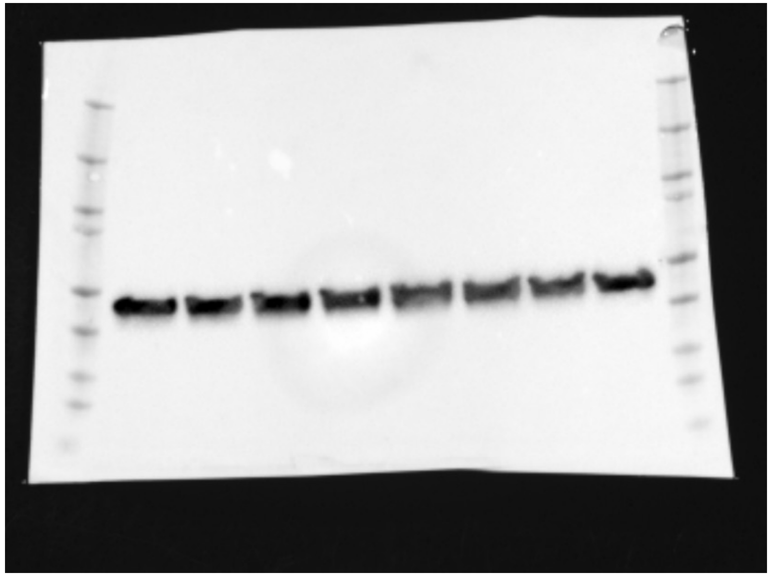

Note: n1 samples ran twice

Full unedited gel for Suppl. Figure S3C

BT474 – Knockdown efficiency of DAB2IP via shRNA

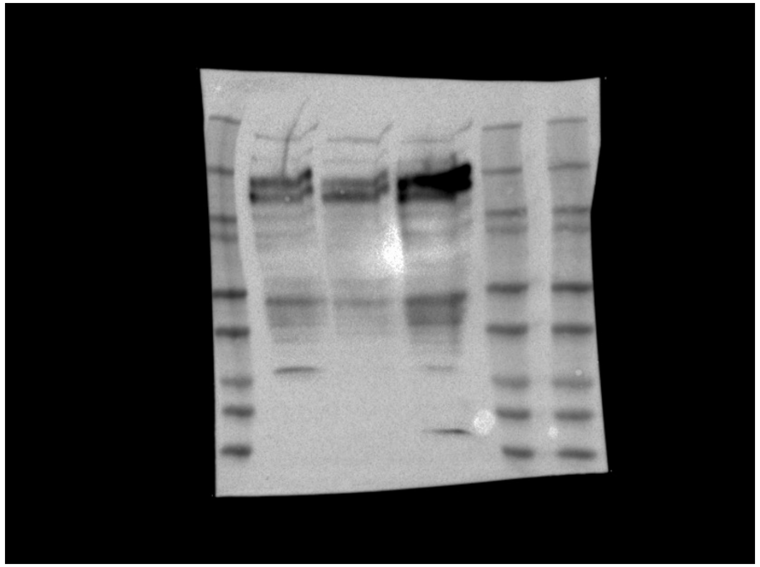

Anti-DAB2IP

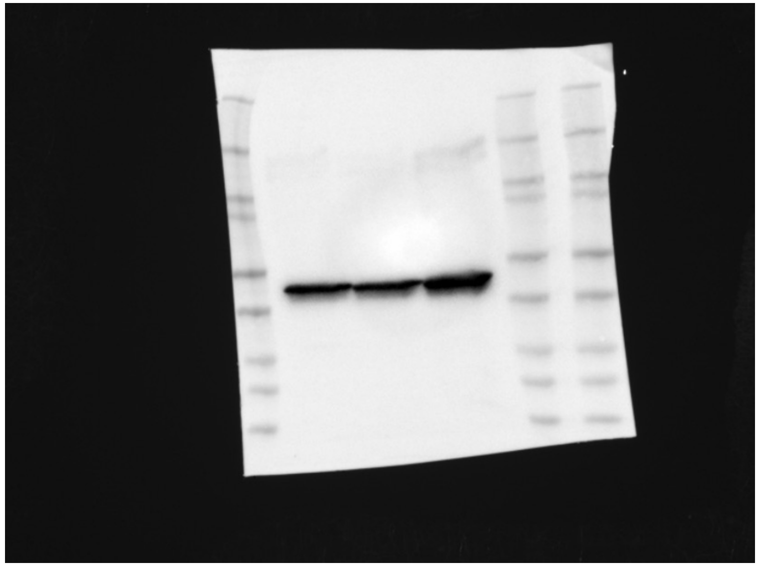

Anti-β-actin

Full unedited gel for Suppl. Figure 5E

Increased expression of Phospho-p38 with decrease in DAB2IP

Anti-DAB2IP

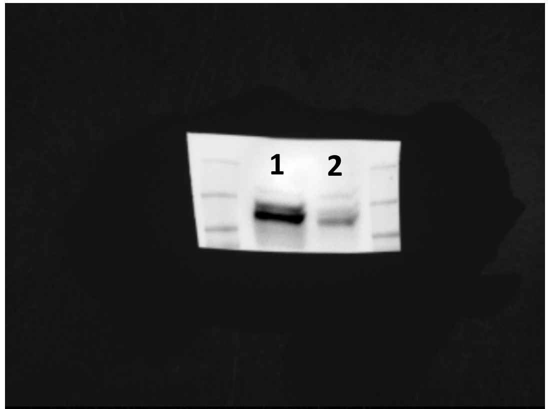

Phospho-p38

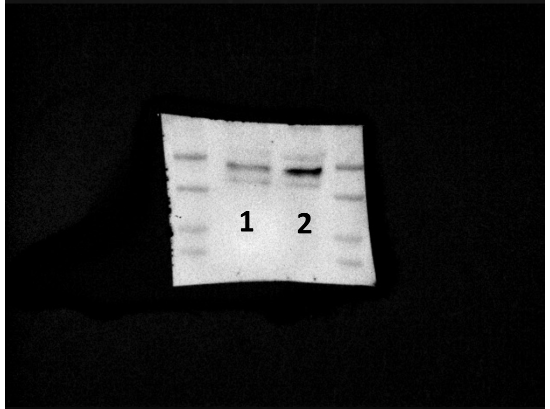

Anti-β-actin

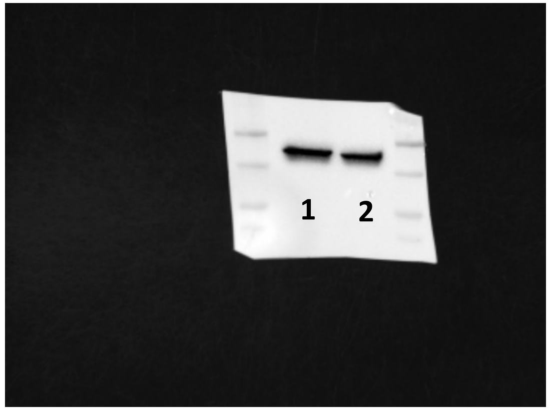

Lane 1: siControl
Lane 2: siDAB2IP

Total p38

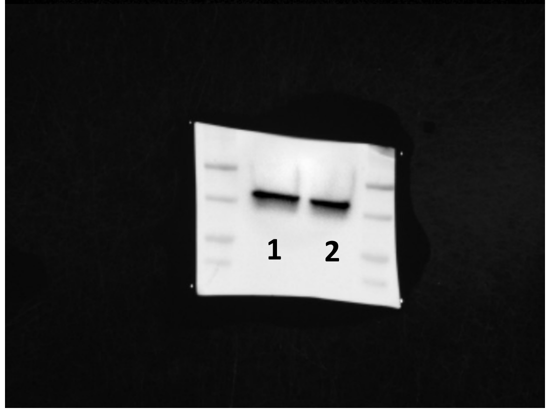

Full unedited gel for Suppl. Figure S8A

T47D – Knockdown efficiency of DAB2IP, BIRC5 via siRNA.

Blot was cut for incubation in antibodies

Lane 1: siControl

Lane 2: siDAB2IP

Lane 3: siBIRC5

Lane 4: siDAB2IP + siBIRC5

Anti-DAB2IP

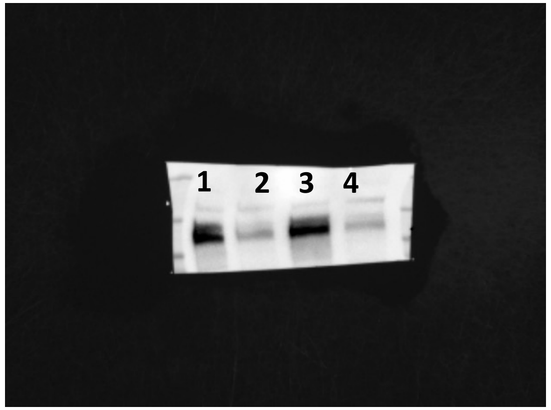

Anti-BIRC5

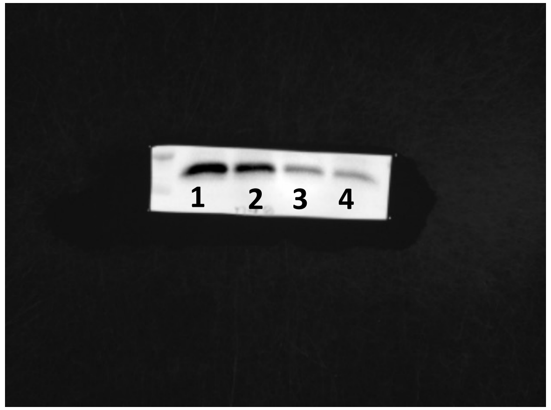

Anti-β-actin

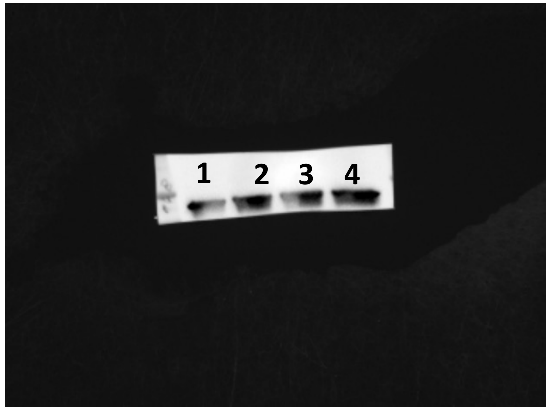

Supplement: Unedited blot and gel images [file jciinsight-9-171705-s052.pdf]
